# Surfactant-Enhanced In Situ Remediation Beneath an Occupied Residential Building: Reduction of the BTEX Vapor-Intrusion Source Term and Field Validation in a Single Field Season

**DOI:** 10.3390/toxics14100906

**Published:** 2026-10-14

**Authors:** Gautier Decabooter

**Affiliations:** Biotix Inc., Quebec City, QC G1N 2E3, Canada; gdecabooter@biotix.ca

**Keywords:** petroleum hydrocarbons, BTEX, soil flushing, in situ remediation, biodegradable surfactant, vapor intrusion, risk-based regulatory compliance, field-scale, occupied building

## Abstract

Petroleum hydrocarbon contamination beneath occupied buildings poses a chronic BTEX vapor-intrusion risk, yet field-scale evidence for in situ remediation under occupied residential structures remains scarce. We report a full-scale surfactant-enhanced remediation (SER) campaign at a former service-station site in Quebec City (Canada), where 235 m^2^ of contaminated soil extended directly beneath an occupied multi-unit residential building. Through 46 dual-action injection–extraction wells, 15.3 m^3^ of a biodegradable polysorbate-family washing solution and 31.2 m^3^ of rinse water were delivered by a stop-and-go protocol over 115 days. Pre-treatment soils reached Criterion C for BTEX (xylenes up to 335 mg kg^−1^). Validation sampling at three sectors showed compliance with Quebec Criterion B at two of them, with a single residual xylenes exceedance (9.0 mg kg^−1^; Criterion B = 5) at Sector 1. Sector-mean abatement reached >99% for ethylbenzene and xylenes at Sector 2, 79% and 90% at Sector 1, and 60–61% for TPH C_10_–C_50_. All validation results complied with the provincial criterion for the methylnaphthalenes, yet 1-methylnaphthalene remained 2.2–5.0 times its inhalation-driven USEPA residential screening level. Biodegradable SER can substantially reduce the BTEX vapor-intrusion source term within a single season without displacing occupants; occupant exposure itself was not measured.

## 1. Introduction

Petroleum hydrocarbons (PHCs) remain among the most widespread soil and groundwater contaminants worldwide, the legacy of decades of fuel storage, distribution, and industrial activity [1,2]. Their persistence in the subsurface poses long-term toxicological and ecotoxicological risks: aromatic monocyclic compounds (BTEX—benzene, toluene, ethylbenzene, xylenes) are mobile, volatile, and acutely toxic, with benzene classified by IARC as carcinogenic to humans (Group 1) [3] and toluene/xylenes implicated in ototoxicity and central-nervous-system depression [4]; heavier C_10_–C_50_ aliphatic and aromatic fractions exhibit low solubility, high sorption, and very slow natural attenuation [5,6]. When such contamination occurs beneath occupied buildings, vapor intrusion of BTEX into indoor air becomes a continuing exposure pathway requiring active management [7].

In Quebec (Canada), management of petroleum-contaminated soils is governed by the Land Protection and Rehabilitation Regulation (*Règlement sur la protection et la réhabilitation des terrains* (RPRT, RLRQ c. Q-2, r. 37)) and the Soil Protection and Contaminated Land Rehabilitation Policy (*Politique de protection des sols et de réhabilitation des terrains contaminés*) [8,9]. Generic Criterion B—700 mg kg^−1^ for total petroleum hydrocarbons in the C_10_–C_50_ range (TPH C_10_–C_50_; referred to in the Quebec regulatory framework as *hydrocarbures pétroliers*, HP C_10_–C_50_) in residential land use—constitutes the legal remediation threshold [10], with attainment certified by an accredited expert. Soil remediation is regulated at the provincial level in Canada; the soil quality guidelines of the Canadian Council of Ministers of the Environment (CCME) are national benchmarks that provinces may adopt or adapt, and they are compared with the Quebec generic criteria for the parameters of this study in Table S8 [2,11]. Sites exceeding Criterion B therefore require demonstrably effective remediation strategies whose performance is documented against this regulatory benchmark and, increasingly, against toxicological reference values such as the USEPA IRIS reference concentration for benzene (3 × 10^−2^ mg m^−3^) [12].

Conventional remediation methods—excavation, pump-and-treat, soil vapor extraction, thermal desorption, and chemical oxidation—face substantial constraints at urban confined sites [13,14]. Excavation is impractical when contamination underlies an occupied structure; pump-and-treat is inefficient against strongly sorbed PHCs and may run for decades [5,15]; thermal and oxidative methods are intrusive, energy-intensive, and often unsafe in residential settings [13,14]. Surfactant-enhanced remediation (SER) addresses these limitations by reducing interfacial tension, forming micelles that solubilize hydrophobic contaminants, and promoting desorption from the soil matrix [16,17,18,19]. Recent reviews and a comprehensive Toxics state-of-knowledge synthesis highlight SER as a leading in situ technology for petroleum source-zone remediation [20,21,22], and the remediation of contaminated soil and groundwater has recently been identified as a research priority [23], yet peer-reviewed field-scale demonstrations beneath occupied residential structures remain almost absent: most reported field studies have been conducted at decommissioned industrial sites or military bases [24,25,26,27]. To the author’s knowledge, only McGee [28] (electro-thermal beneath an occupied apartment) and Martel et al. [29] (PCB micellar flushing beneath an industrial building) have documented in situ chemical/thermal flushing performed under operational structures.

This study addresses that gap. We report a complete field-scale SER campaign performed at a residential site in Quebec City where 235 m^2^ of PHC-contaminated soil extended directly beneath the footprint of an actively occupied multi-unit residential building. The objectives are to: (1) evaluate the field-scale performance of a biodegradable polysorbate-family surfactant–co-solvent flushing solution for BTEX and TPH C_10_–C_50_ removal; (2) quantify the toxicological risk reduction by comparing pre- and post-treatment concentrations to both regulatory criteria and human-health reference values; (3) demonstrate the operational feasibility of completing a full SER cycle—including regulatory validation—within a single field season beneath an occupied structure; and (4) provide transparent operational parameters (flow rates, surfactant volumes, blower capacity, validation statistics) so the work can be reproduced and benchmarked.

## 2. Materials and Methods

### 2.1. Site Description and Historical Context

The study site is located in an urban residential area of Quebec City, Quebec, Canada. The property covers approximately 4100 m^2^ and is occupied by a multi-unit residential building covering ≈70% of the lot. The site is zoned residential and is surrounded by residential land use. Prior to residential redevelopment, the site operated as a service station between 1946 and 1974. Former infrastructure included a fuel-pump island, an underground storage tank (UST) pit, a hydraulic lift, an oil–water separator, and a paint room. Phase I and Phase II environmental site assessments conducted in 2024 by an independent environmental consulting firm (unpublished commercial reports, data on file with the corresponding author) identified subsurface PHC contamination attributable to former UST operations. The contaminated zone of interest covers 235 m^2^: ≈152 m^2^ in exterior lawn and garden areas adjacent to the building and 83 m^2^ directly beneath the existing building footprint (Figure 1). Organoleptic indicators (visual staining and petrochemical odor) were observed from 3.0 to 4.5 m below ground surface (bgs), corresponding to the capillary fringe and saturated zone.

**Figure 1:**
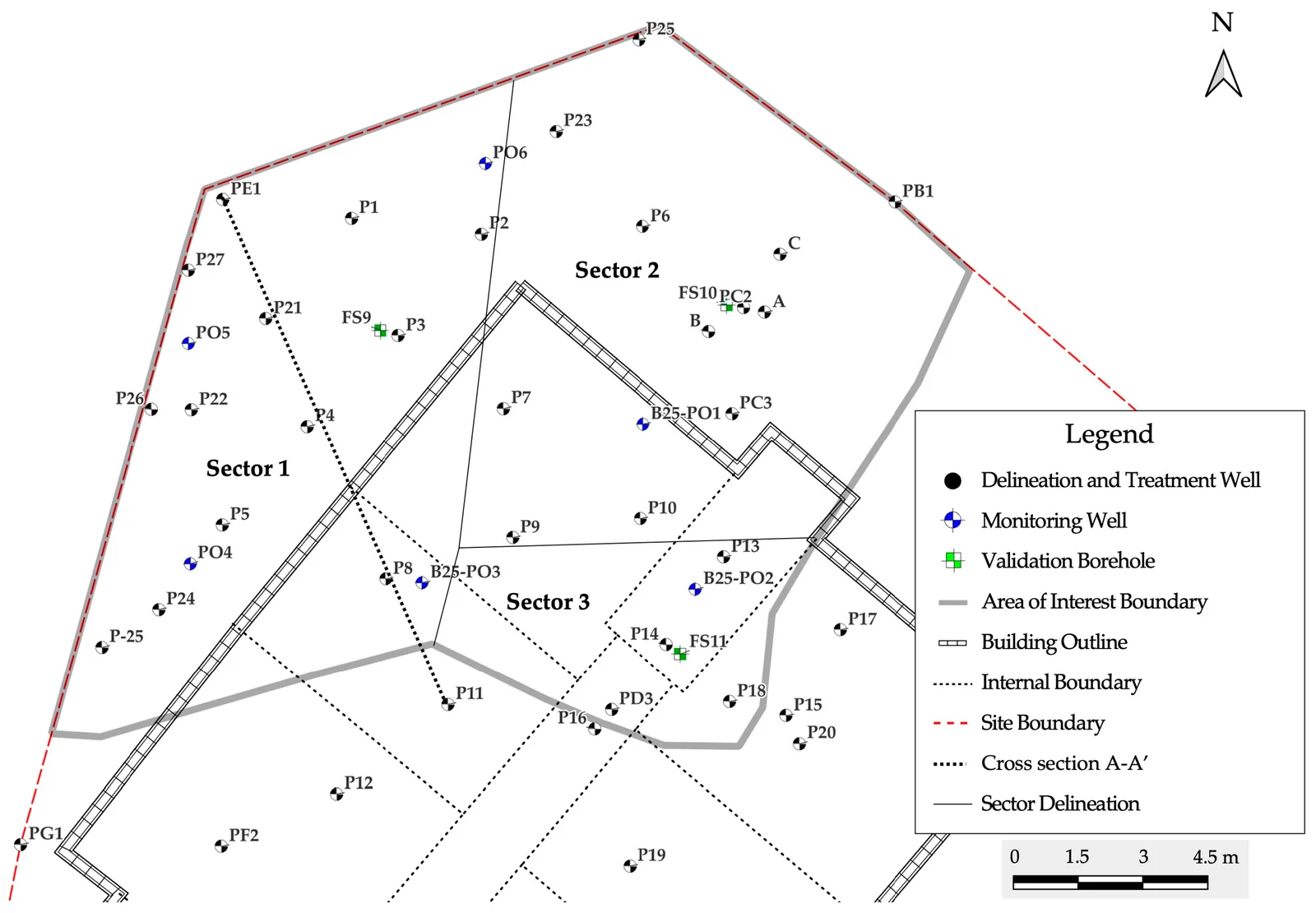
Site layout and well network. Plan view of the 235 m^2^ area of interest (grey outline) at the former service-station site, showing the three validation sectors delineated during the Phase II/III investigations (Sector 1, 110 m^2^; Sector 2, 100 m^2^; Sector 3, 25 m^2^; thin black lines). Black circles: delineation boreholes and dual-action injection–extraction wells (P-series), installed by direct-push drilling and screened over 3.0–4.5 m bgs. Blue circles: pre-existing monitoring wells retained for hydraulic monitoring (PO- and B25-PO series). Green squares: post-treatment validation borehole clusters (FS9, FS10, FS11; 5 December 2025). Wells A, B, and C, located 0.5, 1.0, and 1.5 m from injection well PC2, are the observation points of the 4–7 March 2025 injection–diffusion test used to establish the 1.5 m effective injection radius (Section 2.2.2). Hatched line: footprint of the occupied multi-unit residential building; dotted lines: internal partition walls; red dashed line: property boundary. Grid north at top. Trace A–A′ indicates the cross-section shown in Figure 2.

The site lies on flat ground in the St. Lawrence Lowlands, under a humid continental climate (Köppen Dfb) [30]. Climate normals for Québec City (Jean Lesage International Airport, 1981–2010) give a mean annual air temperature of 4.2 °C and a mean annual precipitation of 1190 mm, including 303 cm of snowfall [31]. Surficial deposits are mapped as undifferentiated alluvium overlying sedimentary rocks of the St. Lawrence Platform (shale, slate, limestone, dolomite, and sandstone) [32]; bedrock was not reached by any borehole (deepest 7.5 m). About 80% of the lot is sealed (building ≈ 70%, asphalt parking ≈ 10%; lawns ≈ 20%), and runoff from sealed surfaces is collected by the municipal storm sewer; infiltration is therefore limited to the lawn areas, which include ≈152 m^2^ of the 235 m^2^ treated area. The local hydraulic gradient is low (≈1.3 × 10^−3^, October 2024), and groundwater at the site is not used for water supply, the area being served by the municipal network.

Phase II drilling (June–July 2024; boreholes TF-24-01 to TF-24-09) identified PHC contamination in the capillary fringe and saturated zone, with TPH C_10_–C_50_ exceeding Quebec generic Criterion B for residential use [10]. Subsequent Phase III delineation (September–October 2024), SER installation drilling (March–April 2025), and in-SER monitoring (September–October 2025) extended this dataset to 43 samples across 40 boreholes (Table S1, Supplementary Materials); the resulting contamination inventory is reported in Section 3.2. Excavation was excluded as a remediation option due to the presence of the occupied multi-storey building directly above the primary source zone.

### 2.2. Hydrogeological Characterization

#### 2.2.1. Hydraulic Head, Conductivity, and Soil Properties

Hydraulic head was monitored using six screened wells. Water levels were recorded with two portable electric interface probes (±5 mm) and corrected for tidal and barometric variations. Saturated hydraulic conductivity (*K*) was determined by constant-head Lefranc tests per BNQ 2501-135 [33] in five wells. Grain-size distribution was determined by wet sedimentation following CEAEQ MA. 100—Gran. 2.0 [34], independently characterizing aquifer texture and validating *K* by Hazen’s empirical law [35], the known limitations of which [36] are acknowledged; Hazen estimates were used only as a cross-check on the Lefranc results.

Borehole logs from the Phase II–III and well-installation campaigns show a consistent sequence across the area of interest: (i) heterogeneous anthropogenic fill of sand and gravel with traces of organic matter and residual materials (locally 2–5% coal and ash debris), generally 0.3 m thick under the lawns and up to ≈2.1 m beneath the building slab; (ii) where present, a grey clay to clayey-silt layer 0.5–2.0 m thick, of lower permeability; and (iii) brown fine silty sand turning grey with depth, which extends to the base of all boreholes. The upper profile was excavated and backfilled during the service-station and residential-development phases, so the site does not carry an undisturbed pedological profile and no taxonomic soil classification was performed. The treated interval (3.0–4.5 m bgs) lies predominantly within unit (iii), below the solum, in the capillary fringe and upper saturated zone; it is therefore described here as an aquifer sediment rather than as a genetic soil horizon.

Within the treated interval, the composite sample contained 93.7% sand, 6.0% silt + clay, and 0.3% gravel (Section 3.1), and moisture contents in the post-treatment cores were 14.4–17.7% (Table S3). Porosity, bulk density, pH, organic carbon, cation exchange capacity, specific surface area, and water repellency were not measured; the effective porosity of 0.30 used for pore-volume normalization (Section 2.5) is a typical value for this texture, the total porosity of sands ranging from 0.25 to 0.50 [37]. Pre-treatment groundwater chemistry (petroleum hydrocarbons, BTEX, naphthalene, nutrients, and field pH) is reported with the soil properties in Table S7; no light non-aqueous phase liquid was detected with the interface probe, and dissolved oxygen, oxidation–reduction potential, and temperature were not recorded under undisturbed conditions.

#### 2.2.2. Field Diffusion Test and Effective Injection Radius

A field injection–diffusion test was conducted on 4–7 March 2025 to determine the effective injection radius. The washing solution was injected via well PC2 at 7.5 L min^−1^ over 2 h. Hydraulic response was monitored using pressure leveloggers at 0.5 m (well A), 1.0 m (well B), 1.5 m (well C), and 2.0 m (well PO2). Water-table rise was +40 cm at 1.0 m and +8.7 cm at 1.5 m, with negligible response beyond 2.0 m. Physicochemical monitoring (pH, salinity) confirmed solution arrival at 1.0 m within 24 h. A 1.5 m design injection radius was therefore adopted, ensuring overlap between adjacent well influence zones while remaining conservative. Capture-zone overlap at the adopted 1.5 m radius was verified with a three-dimensional groundwater flow model built in FEFLOW 7.0 (DHI, Hørsholm, Denmark) [38] discretized into three layers consistent with the stratigraphy of Figure 2, with layer-wise *K* assigned from the Lefranc tests and grain-size analyses (arithmetic mean 4.24 × 10^−5^ m s^−1^). The model was used solely as a qualitative design-support tool; it was not calibrated, no calibration statistics are reported, and no quantitative claim in this study rests on its output.

### 2.3. Feasibility Testing

#### 2.3.1. Laboratory Column Testing

The washing solution was first validated in laboratory column experiments. Washed and dried sand (grade 00; 120 g) was artificially contaminated with diesel fuel to yield an initial TPH C_10_–C_50_ concentration of 7080 mg kg^−1^ dry weight, corresponding to residual adsorbed-phase contamination only (no free-phase liquid). Three replicate columns were operated at a constant flow of 14 mL min^−1^: three pore volumes of washing solution were injected, followed by two pore volumes of tap-water rinse. Two acceptance criteria governed transfer to field deployment: (i) TPH C_10_–C_50_ abatement ≥ 90% under controlled conditions, and (ii) practical deployability at full scale, assessed qualitatively against alternative remediation chemistries on three grounds—reagent cost per pore volume, feasibility of on-site preparation using municipal water and non-specialized equipment, and treatability of the resulting effluent by the on-site aeration train alone. Absolute cost data are commercially confidential and are not reported; criterion (ii) was applied as an internal relative comparison. Column outcomes against both criteria are reported in Section 3.1.

#### 2.3.2. Field Demonstration Test

Prior to full-scale implementation, a field demonstration test was conducted in September 2024. Approximately 3600 L of washing solution followed by 3200 L of water rinse were injected via well PC3. Contamination reductions were verified through four validation boreholes (FV1–FV4) and supported full-scale deployment.

### 2.4. System Design and Well Installation

Based on the 1.5 m effective injection radius (Section 2.2.2), 46 dual-action treatment wells were installed by direct-push drilling, each capable of both injection and extraction (Figure 1). Pre-existing observation wells (PO-series) were retained for hydraulic monitoring. The well network was distributed across two access zones: 26 exterior wells in lawn/garden areas and 20 interior wells installed beneath the building footprint through core drilling of the reinforced concrete slab using a small-footprint rotary rig compatible with confined indoor access. All wells were screened at the depth interval corresponding to the capillary fringe and saturated zone (3.0–4.5 m bgs) (Figure 2). Well installation followed the procedure described below.

#### Borehole Installation and Soil-Core Collection

Boreholes were drilled with a Geoprobe^®^ 420 M (interior, track-mounted) or Geoprobe^®^ 6620DT (exterior, crawler-mounted; Geoprobe Systems, Salina, KS, USA) direct-push rig, without drilling fluids. Continuous cores were retrieved at 0.5 m intervals or at each stratigraphic change. Soil samples for TPH C_10_–C_50_ and PAH analyses were collected in 250 mL amber glass jars per MDDEP Cahier 5 [39]. VOC field screening used sealed plastic bags read within 1 h on a PID Tiger (ION Science, Royston, UK; calibrated with isobutylene; LOD 1 ppb). The interval with the highest VOC reading per borehole was selected for discrete BTEX laboratory sampling using an En Novative Technologies sampler with single-use EnCore™ (En Novative Technologies, Green Bay, WI, USA) capsules; capsules were iced and shipped within hold times per DR-09-12. Groundwater levels and free-phase hydrocarbons were checked with a Solinst MINI 112 (Solinst Canada Ltd., Georgetown, ON, Canada) interface probe before each campaign, and in situ T, pH, ORP, and EC were recorded with a Hanna HI 9829 (Hanna Instruments, Woonsocket, RI, USA) multi-parameter probe per MDDEP Cahier 3 [40].

Each borehole was converted to a dual-action well (injection/extraction). Interior wells used 25.4 mm (1″) PVC casing with a 1.5 m slotted screen; exterior wells used 38.1 mm (1.5″) PVC casing with the same screen length. All wells were sealed with a bentonite–cement plug to prevent vertical migration and surface-water intrusion.

### 2.5. Remediation Operation

The remediation campaign was conducted from 12 August to 5 December 2025. The treatment system is shown schematically in Figure 3. During extraction phases, groundwater, mobilized hydrocarbons and soil gas were withdrawn from the dual-action wells by a vacuum pump (vacuum-enhanced, multi-phase extraction). The washing solution was applied via a stop-and-go injection–extraction protocol to maximize contact time between the solution and adsorbed PHCs. Three dedicated injection wells (P23, P18, PC2) each received 3.6 m^3^ of washing solution followed by 3.6 m^3^ of water rinse. Subsequent stop-and-go cycles alternated diluted washing solution and water rinse across all treatment wells, progressively displacing the washing front. In total, 15.3 m^3^ of washing solution and 31.2 m^3^ of rinse water were injected (cumulative injected fluid = 46.5 m^3^). Normalized to the pore volume of the treated interval (235 m^2^ × 1.5 m screened thickness × an assumed effective porosity of 0.30 = 105.8 m^3^), this corresponds to 0.14 pore volumes (PV) of washing solution, 0.30 PV of rinse water, and 0.44 PV of total injected fluid. Because porosity was not measured, this normalization is approximate: effective porosities of 0.25–0.40 would give pore volumes of 88–141 m^3^ and washing-solution doses of 0.11–0.17 PV (Table S6), which does not change the comparison with published field campaigns in Section 4.1. Extracted groundwater was continuously conveyed to the effluent treatment system (Section 2.6).

### 2.6. Effluent Treatment

All extracted groundwater was routed to the treatment train; groundwater chemistry is therefore reported through the extracted-water monitoring described below. Extracted groundwater was collected in a 10,000 L primary tank and treated by diffused-air aeration. Untreated water withdrawn from extraction wells in the BTEX source zone contained 0.7–1.8 mg L^−1^ total BTEX on 28 August 2025 (wells P24 and P5) and 1.8–7.5 mg L^−1^ on 26 September 2025 (wells P4, P5, P8, P21, and P22); after aeration, all treated batches met the discharge limits (Section 3.4).

The aeration unit consisted of a rectangular tank fitted with a network of perforated plastic pipes connected to a mechanical blower (rated capacity 0.26 m^3^ s^−1^ (550 CFM); discharge pressure 103 kPa (15 PSI)). Fine bubbles were generated by forcing compressed air through the pipe-network perforations, producing a high air–water interfacial area; volatilization was driven by Henry’s law. BTEX compounds are particularly amenable to this treatment owing to their high Henry’s law constants (*H* = 5.5–7.9 × 10^−3^ atm m^3^ mol^−1^ at 25 °C, i.e., dimensionless equivalents *H*′ = 0.22–0.32) [41,42]; lighter aliphatics (C_10_–C_16_) are also amenable, while heavier fractions (C_16_–C_50_) are not significantly removed. Treated water was transferred to a 10,000 L compliance-verification tank and then released into the City of Quebec combined sewer under a municipal discharge permit, including a maximum authorized discharge rate of 6.94 L min^−1^.

### 2.7. Effluent Monitoring and Discharge Compliance

Effluent quality was monitored through 10 discrete events between 14 August and 28 November 2025, sampled at the compliance-verification tank downstream of the aeration unit. Benzene, toluene, ethylbenzene, xylenes, naphthalene, and TPH C_10_–C_50_ were determined at each event and compared with the municipal sewer discharge limits (Table S5). Results are reported in Section 3.4.

Extracted groundwater was not metered by a cumulative flow totalizer. The cumulative volume pumped, treated, and discharged over the campaign was instead compiled from the site operating records at approximately 810 m^3^, equivalent to about 81 fillings of the 10 m^3^ compliance-verification tank. This figure is consistent with the extraction rate measured at start-up (≈10 m^3^ per 24 h of pumping) and with the upper bound set by the discharge permit (6.94 L min^−1^ over 115 days ≈ 1150 m^3^). The extracted volume therefore exceeded the injected volume (46.5 m^3^; Section 2.5) by a factor of about 17. Pressure-transducer records at three observation wells (November 2025) showed drawdowns of 10–12 cm developing over ≈15 h of pumping and recovering over ≈17 h after pumping stopped. These data indicate that net gradients were directed toward the extraction wells during extraction phases; they do not exclude transient outward gradients during injection sequences, when water levels rose locally (Section 2.2.2).

### 2.8. Validation Sampling

Validation drilling was conducted on 5 December 2025 using the same direct-push procedure as in Section 2.4, in order to confirm compliance with the Soil Protection and Contaminated Land Rehabilitation Policy [9] and the RPRT [8]. Sampling was distributed across the three contamination sectors (Figure 1) delineated during Phase II/III investigations (Sector 1: 110 m^2^; Sector 2: 100 m^2^; Sector 3: 25 m^2^) per the Field Characterization Guide [43]. At Sectors 1 and 2, three closely spaced boreholes (separation < 0.3 m) were drilled per sector; at Sector 3, whose area is 25 m^2^, a single borehole was drilled and a field duplicate collected. Within each borehole, continuous soil cores were retrieved over the target depth interval (capillary fringe and saturated zone, 3.0–4.5 m bgs) and screened at successive depth increments for volatile organic compounds using a calibrated PID (Tiger, ION Science; calibrated with isobutylene; LOD 1 ppb). The increment(s) yielding the highest PID response—interpreted as the most contaminated horizon(s)—were selected for laboratory analysis (TPH C_10_–C_50_, BTEX, PAH). The validation campaign yielded seven discrete soil samples: three at Sector 1 (FS9A-E2, FS9B-E7, FS9C-E4), three at Sector 2 (FS10A-E4, FS10B-E2, FS10C-E3), and one at Sector 3 (FS11-E4), one PID-selected sample per borehole. BTEX were additionally determined in analytical triplicate on five of these samples and a field duplicate was collected at FS11-E4, giving 16 BTEX, 5 TPH C_10_–C_50_, and 8 PAH determinations in total. Replicate determinations are treated as measurement replicates of a single sample and are averaged to the sample level before any statistic is computed; they are not counted as independent observations. Per-sample analytical detail is provided in Table S3. Validation borehole locations were constrained by physical access. The direct-push rig requires clearance that was unavailable at several of the most contaminated characterization points: P8 lies within an occupied dwelling unit that could not be entered for drilling; P26 and P27 are within the setback of the property boundary; and mature trees restricted access in the exterior lawn areas. Validation was therefore performed at the accessible locations within each of the three sectors delineated during the Phase II/III investigations, consistent with the sector-based compliance approach of the Field Characterization Guide [43]. Compliance is thus demonstrated at the sector scale rather than at every individual pre-treatment sampling point.

### 2.9. Chemicals and Washing-Solution Composition

All chemical reagents were purchased from commercial suppliers. The washing solution was prepared on site by diluting a concentrated proprietary blend with municipal tap water in a tote tank. The formulation contains four functional components: (i) a non-ionic surfactant of the polysorbate family at a working concentration of 2% (*v*/*v*); (ii) two co-solvents of the glycol-ether family—one short-chain and one medium-chain—which enhance contaminant desorption and reduce micellar viscosity; (iii) an inorganic electrolyte (alkali-metal chloride) modulating the critical micelle concentration (CMC); and (iv) an inorganic phosphate base adjusting pH to favor surfactant performance and subsurface transport. The polysorbate-family non-ionic surfactant was selected for its food-grade certification, its well-documented aerobic biodegradation in soil and groundwater [44], and the favorable performance of polysorbate-family non-ionic surfactants reported in field- and laboratory-scale source-zone remediation [22,27,45]. Acute effects on plankton growth and survival for this surfactant class are reported at concentrations of the order of 100 mg L^−1^, but the same work reports sublethal responses at 10 mg L^−1^ and community-level responses one to two orders of magnitude lower still [46]; residual surfactant reaching surface waters is therefore treated as a design constraint rather than a negligible term (Section 4.3). Its reported acute aquatic toxicity is likewise low: a MICROTOX^®^ EC_50_ of 7.0 g L^−1^ (95% confidence interval 4.7–10.5 g L^−1^) classifies this surfactant as practically non-toxic to aquatic organisms under the USEPA ecological risk framework [47], several orders of magnitude above the residual concentrations expected after rinse. The specific surfactant identity, concentrate manufacturer, and exact blend ratios are withheld under a confidentiality agreement; full QA/QC documentation is available from the corresponding author upon reasonable request and execution of a non-disclosure agreement. A total of 15.3 m^3^ of washing solution and 31.2 m^3^ of rinse water (combined 46.5 m^3^) was injected over the campaign.

### 2.10. Analytical Methods

All analyses were performed by an external laboratory accredited by the Quebec Ministry of Environment, Climate Change, Wildlife and Parks (Ministère de l’Environnement, de la Lutte contre les changements climatiques, de la Faune et des Parcs (MELCCFP)), following CEAEQ standardized protocols. QA/QC included method blanks, field duplicates, matrix spikes, and certified reference standards per analytical batch. TPH C_10_–C_50_ was quantified by GC–FID per CEAEQ MA. 400—HYD 1.1 (Rev. 9, 27 January 2025): hexane liquid–solid extraction, silica-gel cleanup, GC–FID against certified petroleum hydrocarbon standards; results in mg kg^−1^ dry weight; comparison thresholds: A = 100, B = 700, C = 3500 mg kg^−1^. PAHs (naphthalene + 15 USEPA priority compounds) were determined by GC–MS in SIM mode per CEAEQ MA. 400—HAP 1.1 (Rev. 14, 30 October 2024); naphthalene was compared to RPRT Criterion B = 5 mg kg^−1^. VOCs/BTEX were analyzed by purge-and-trap GC–MS per CEAEQ MA. 400—COV 2.0 (Rev. 1, 2 October 2024), with sample purging at 40 °C for 11 min, multi-sorbent trapping (Tenax/silica gel/charcoal), and dual full-scan/SIM detection.

### 2.11. Statistical Analysis and Risk Framing

Post-treatment soil concentrations of TPH C_10_–C_50_ at validation sectors are reported as mean ± standard deviation (*n* ≥ 3) or as individual values (*n* < 3). Compliance with Quebec Criterion B (700 mg kg^−1^ for residential land use) is established primarily on individual sample results, as required by the provincial framework: every validation result must lie below the criterion. Sector-scale central tendency is reported in support, as the mean and the one-sided 95% upper confidence limit (UCL) of the mean, computed following USEPA guidance for environmental compliance evaluation [48]. Non-detects are substituted at the full reporting limit throughout, yielding an upper-bound estimate. Analytical replicates (laboratory duplicate and triplicate determinations on a single soil sample) and field duplicates are treated as measurement replicates and are averaged to the sample level before any statistic is computed; they are not counted as independent observations. Given sample sizes of *n* = 3 (FS9, FS10) and *n* = 1 plus field duplicate (FS11), inferential testing is reported for FS10 only and is regarded as indicative rather than confirmatory. For FS9, TPH C_10_–C_50_ was not analyzed post-treatment because pre-treatment concentrations at this sector were already below Criterion B (226–351 mg kg^−1^ at P3); remediation at this sector was driven by ethylbenzene (7.4 mg kg^−1^) and xylenes (36.1 mg kg^−1^), both above Criterion B (5 mg kg^−1^), which were therefore prioritized for post-treatment analysis. Toxicological context was provided by comparing post-treatment soil concentrations to USEPA regional screening levels for residential soil [49] and to the USEPA IRIS reference concentration for benzene [12].

## 3. Results

### 3.1. Hydrogeological Characterization and Feasibility Testing

Lefranc injection tests yielded *K* values from 2.66 × 10^−5^ to 6.35 × 10^−5^ m s^−1^ (arithmetic mean 4.24 × 10^−5^ m s^−1^). These values were consistent with grain-size estimates by Hazen’s law. The composite soil sample comprised 93.7% sand, 6.0% silt + clay, and 0.3% gravel, classifying the matrix as a fine-to-medium sand on the USDA textural triangle. The field injection–diffusion test confirmed an effective 1.5 m injection radius, leading to the 46-well layout described in Section 2.4. Column experiments confirmed mean TPH C_10_–C_50_ removal of 95.7% (range 95.3–95.9%; *n* = 3) under controlled conditions, providing proof-of-concept for adsorbed-phase mobilization in a sandy matrix. The September 2024 field demonstration (3600 L washing solution; 3200 L rinse via PC3) was validated through four boreholes (FV1–FV4), confirming transferability of laboratory results to field conditions.

### 3.2. Pre-Remediation Contamination

TPH C_10_–C_50_ concentrations ranged from below the 100 mg kg^−1^ reporting threshold to 1780 mg kg^−1^, with 27 of 43 samples above the reporting limit. Classified on their most restrictive parameter, 10 samples were at or below Criterion A, 7 in the A–B range, 12 in the B–C range, and 14 above Criterion C. The >C classifications were driven by BTEX at 13 samples—principally xylenes (up to 335 mg kg^−1^) and ethylbenzene (up to 73.9 mg kg^−1^), concentrated in the source zone—and by 2-methylnaphthalene at one (PC3, 11.4 mg kg^−1^ against a Criterion C of 10 mg kg^−1^); no sample exceeded Criterion C for TPH C_10_–C_50_. Five samples exceeded Criterion B for TPH C_10_–C_50_ (maximum 1780 mg kg^−1^ at PC3), naphthalene exceeded its Criterion B of 5 mg kg^−1^ at six samples (maximum 10.8 mg kg^−1^ at P2), and benzene exceeded its Criterion B of 0.5 mg kg^−1^ at two (P8, 0.6; P27, 0.8 mg kg^−1^). The methylnaphthalene isomers, each subject to a Criterion B of 1 mg kg^−1^, were the most frequently exceeded parameter group at the site: 23 of the 43 samples exceeded Criterion B for at least one of 1-methylnaphthalene, 2-methylnaphthalene, and 1,3-dimethylnaphthalene, with maxima of 5.0, 11.4, and 2.3 mg kg^−1^, respectively, all at PC3. The spatial distribution of contamination above Criterion B is shown in Figure 4. The maps in Figure 4 were produced by ordinary kriging of the characterization dataset (Table S1) and are presented as a qualitative illustration of the spatial distribution of contamination. Given the sample density (43 samples over 235 m^2^) and the short-range heterogeneity documented by field duplicates (relative percent differences of 55–66%; Tables S2 and S4), the interpolated surfaces should not be read as quantitative concentration estimates at unsampled locations, and no result reported in this study derives from them. A total of 6 of these 43 samples (PO4, PO5, PO6, P26, P27, and the second P25 sample) were collected during the active remediation phase in September and October 2025 rather than before it (campaign code MON, Table S1); they are retained because they characterize parts of the source zone not otherwise sampled, but they do not constitute a strictly pre-treatment baseline.

### 3.3. Treatment Performance and Validation

Post-remediation validation drilling on 5 December 2025 sampled three sectors (FS9, FS10, FS11) covering 235 m^2^. Pre/post comparison at validation sectors is summarized in Table 1.

At Sector 2 (FS10), the three individual TPH C_10_–C_50_ results were 134, 416, and <100 mg kg^−1^, the highest representing 59% of Criterion B; the sector mean was 217 ± 173 mg kg^−1^ (non-detects at the 100 mg kg^−1^ reporting limit), with a one-sided 95% UCL of 509 mg kg^−1^, and a one-sample one-tailed *t*-test against Criterion B was consistent with compliance (*t*(2) = −4.83, *p* = 0.020). All BTEX and PAH at this sector were at or below the reporting limit except in FS10B-E2, the single residual hot spot of the sector, which returned xylenes at 0.3, 1-methylnaphthalene at 0.9, 2-methylnaphthalene at 0.4, and 1,3-dimethylnaphthalene at 0.5 mg kg^−1^—all below Criterion B. At Sector 3 (FS11), the validation sample returned 381 mg kg^−1^ and its field duplicate 192 mg kg^−1^ (relative percent difference 66%), both below Criterion B. At Sector 1 (FS9), naphthalene (0.6 ± 0.3 mg kg^−1^), the methylnaphthalenes, and ethylbenzene (1.6 ± 1.8 mg kg^−1^) were below Criterion B, but xylenes reached 9.0 mg kg^−1^ in FS9A-E2—confirmed at 6.8 and 6.9 mg kg^−1^ in duplicate and triplicate analysis of the same sample—against a Criterion B of 5 mg kg^−1^. Criterion B was therefore met at Sectors 2 and 3 but not at all Sector 1 validation points, where a residual xylenes hot spot persisted despite a 75% reduction relative to the pre-treatment concentration at P3 (36.1 mg kg^−1^). A targeted complementary treatment of this residual zone is scheduled for 2026.

Sector-mean abatement for TPH C_10_–C_50_ was 60% (Sector 2) and 61% (Sector 3). BTEX abatement was higher but strongly sector-dependent: xylenes 90% at Sector 1 and >99% at Sector 2, ethylbenzene 79% and >99%, respectively; naphthalene was reduced by 77% (Sector 1) to >97% (Sector 2). Computed on the least favorable individual sample rather than the sector mean, the corresponding figures are 75% (xylenes, Sector 1), 41% (ethylbenzene, Sector 1), 24% (TPH, Sector 2), and 49% (TPH, Sector 3). Abatement percentages compare a single pre-treatment borehole per sector (P3, P6, P14) with the post-treatment validation samples at the corresponding sector. Pre- and post-treatment sampling points are closely spaced but not co-located—FS9 adjoins P3 and FS11 adjoins P14, whereas FS10 lies approximately 10 m from P6—and, given field-duplicate relative percent differences of 55–66% at this site, the percentages should be read as sector-scale indicators rather than point-scale mass removals.

### 3.4. Effluent Quality and Discharge Compliance

Effluent quality across the 10 monitoring events remained within municipal discharge limits, including the 6.94 L min^−1^ maximum authorized flow rate (Figure 5). Peak BTEX in treated effluent was recorded on 8 September 2025, during the surfactant mobilization phase: toluene 179 µg L^−1^ (limit 200), xylenes 248 µg L^−1^ (limit 370), benzene 40.3 µg L^−1^ (limit 50—site-specific), and ethylbenzene 53.2 µg L^−1^ (limit 160); TPH C_10_–C_50_ remained below the 100 µg L^−1^ reporting limit throughout this first peak. Effluent BTEX did not decline monotonically. A second mobilization peak was recorded on 14 November 2025 (toluene 111 µg L^−1^, xylenes 186 µg L^−1^, ethylbenzene 34.0 µg L^−1^, benzene 18.0 µg L^−1^), reaching 45–75% of the September maxima, with a smaller intermediate response on 17 October. The two peaks differed in composition. TPH C_10_–C_50_ was below the reporting limit at every event up to and including 17 October and was detected only during the second peak—981 µg L^−1^ on 14 November (6.5% of the 15,000 µg L^−1^ permit limit) and 203 µg L^−1^ on 28 November, when BTEX had already returned to near or below analytical detection limits. The first mobilization event therefore recovered monoaromatic compounds essentially alone, whereas the second recovered C_10_–C_50_ fractions as well, with a slower decline consistent with the lower aqueous solubility and stronger sorption of the heavier fractions. This two-peak pattern is consistent with the stop-and-go protocol, in which successive injection sequences mobilized hydrocarbon from different parts of the treatment area rather than progressively depleting a single mobilized pool.

## 4. Discussion

### 4.1. Performance Benchmarking Against Field-Scale SER Studies

When benchmarked against published field-scale surfactant flushing work, the present results are competitive given the additional logistical constraints of operating beneath an occupied structure. Londergan et al. [24] documented one of the first rigorously reported field-scale surfactant-enhanced aquifer remediation (SEAR) trials, at Hill Air Force Base (Utah, USA), recovering chlorinated DNAPL from a heterogeneous alluvial aquifer using an anionic sodium dihexyl sulfosuccinate formulation with isopropanol as co-solvent, at a delivered dose of the order of 2.4 pore volumes. The closest published analogue to the present formulation chemistry is instead the Bachman Road pilot demonstration reported by Abriola et al. [27], in which approximately 68,000 L of a 6% (*w*/*w*) non-ionic polysorbate-family surfactant solution (Tween 80), some 1.5 pore volumes of the treated zone, were delivered to a relatively homogeneous fine-to-medium sand aquifer under a design explicitly aimed at minimizing the injected solution volume—the same surfactant class and the same textural setting as the treated interval of the present site (93.7% sand; Section 3.1). Ritoré et al. [45] recently demonstrated that mixtures of anionic and non-ionic polysorbate-family surfactants can achieve 58–68% hydrocarbon extraction in laboratory soil-washing experiments, with preferential mobilization of lighter and aromatic fractions—mirroring the BTEX-vs.-HP selectivity pattern observed in the present field campaign and supporting the rationale for combining surfactant + glycol-ether co-solvents in the present formulation. Rao et al. [26] demonstrated 85% DNAPL recovery using a food-grade cosolvent under more controlled experimental conditions. Ciampi et al. [50] applied an alkaline desorbing agent (PetroCleanze™) combined with a persulfate-based oxidant (Regenox™) at an industrial site in Italy, using chemically enhanced desorption to extend the reach of an existing pump-and-treat system, and reported a significant recovery of contaminant mass and a reduction of the residual LNAPL load. McGee [28] reported the closest prior analogue to the present setting: an electro-thermal dynamic stripping process (ET-DSP™) combined with high-vacuum dual-phase extraction, applied to ~125 m^2^ of gasoline-impacted soil (~350 m^3^ contaminated) beneath and adjacent to an occupied apartment building, with benzene as the principal contaminant. That campaign recovered 1800 L of petroleum hydrocarbon and 1500 m^3^ of groundwater over 220 days of extraction, and achieved non-detect benzene (<0.02 mg kg^−1^) in all confirmatory soil samples, at a reported cost of ≈USD 80 m^−3^. The endpoint achieved was therefore more complete than in the present study, but at the cost of an entire apartment unit leased and converted into an operational drilling and process base, soil heating to 80 °C beneath a residence, continuous three-phase power delivery, and nearly twice the operating duration. The present campaign achieved Criterion B compliance at two of the three validation sectors in 115 days, without heating, without vacating any dwelling unit, and using a food-grade biodegradable formulation—a materially lower operational and energetic footprint, although the endpoint reached was less complete than in that campaign.

These benchmarks are not directly commensurable: Londergan et al. [24] and Rao et al. [26] report volumetric or mass-based recovery of contaminant from the extracted phase, whereas the present study reports the reduction in residual soil concentration relative to a regulatory threshold. The comparison is therefore offered as an order-of-magnitude context rather than a like-for-like ranking.

The delivered surfactant dose is at the low end of the published field-scale range: 0.14 PV of washing solution here, against approximately 2.4 PV at Hill Air Force Base [24] and approximately 1.5 PV at Bachman Road [27]. Two factors limit the comparison. First, those campaigns targeted mobile DNAPL source zones, whereas the present site carried residual, adsorbed-phase hydrocarbon with no free product detected in any monitoring well (Section 2.4)—a mass distribution requiring desorption rather than bulk NAPL displacement. Second, the stop-and-go protocol (Section 2.5) maximized contact time per unit volume delivered rather than pore-volume throughput. The abatement reported here should therefore be read as specific to a residually contaminated fine-to-medium sand at low pore-volume throughput, and the dose–response relationship in this regime warrants systematic investigation.

The properties of the treated matrix are consistent with this performance. With 93.7% sand and 6.0% fines, the treated interval has a low specific surface area, carried largely by the fine fraction, since clay-sized particles have specific surface areas several orders of magnitude larger than sand grains [51]; sorption sites for hydrophobic compounds are correspondingly limited, which favors their transfer into the micellar phase. Organic carbon was not measured, but it is expected to be low in a grey fine sand below the water table; below an organic-carbon fraction of about 0.1%, sorption of hydrophobic organic compounds is no longer governed by organic matter alone and mineral surfaces contribute significantly [52]. Conversely, the finer lenses and the overlying clay to clayey-silt layer (Section 2.2.1) are the most likely reservoirs of residual, diffusion-limited contamination, and may contribute to the residual xylenes at Sector 1 and to any rebound detected during post-treatment monitoring. Weathered petroleum residues can also render mineral grains water-repellent, which impedes aqueous flushing [53,54]; lowering interfacial tension is the mechanism by which the surfactant counteracts this effect, although water repellency was not measured at this site.

The notably higher BTEX abatement (79% to >99%) relative to total TPH C_10_–C_50_ is consistent with the physico-chemical properties of aromatic hydrocarbons: their higher aqueous solubility and lower molecular weight make them more mobile and more readily displaced by aqueous flushing solutions than heavier C_10_–C_50_ fractions. The dual-action injection–extraction well design, which promoted continuous advective transport, likely enhanced BTEX mobilization preferentially [21].

### 4.2. Reduction of the Vapor-Intrusion Source Term

Post-treatment concentrations fall below the corresponding USEPA residential-soil regional screening levels (RSLs, November 2024 generic table, TR = 10^−6^, HQ = 1) [49] for xylenes (maximum 9.0 mg kg^−1^ against an RSL of 580 mg kg^−1^), benzene, and toluene (all below reporting limits, RSLs 1.2 and 4900 mg kg^−1^) and naphthalene (maximum 0.8 mg kg^−1^, 40% of the 2.0 mg kg^−1^ RSL). Two compounds retain narrower margins. Ethylbenzene reached 4.4 mg kg^−1^ at FS9A-E2, 76% of its 5.8 mg kg^−1^ RSL, which is cancer-based. More notably, 1-methylnaphthalene exceeded its RSL of 0.18 mg kg^−1^ at all three sectors after treatment—0.7, 0.9, and 0.4 mg kg^−1^, i.e., 3.9, 5.0, and 2.2 times the screening level—while remaining below the Quebec generic Criterion B of 1 mg kg^−1^ applicable to this isomer. Sector-mean 1-methylnaphthalene fell from 5.6, 9.4, and 12.2 times the RSL before treatment to 2.6, 2.0, and 1.7 times after it, a 53–86% reduction that nevertheless does not clear the screening level. This divergence deserves emphasis rather than omission: the 1-methylnaphthalene RSL is driven by an inhalation reference concentration of 3 × 10^−6^ mg m^−3^—a recent and deliberately conservative value—and inhalation is precisely the exposure route of concern beneath an occupied building. Regulatory compliance with a generic provincial criterion therefore does not, by itself, establish that the vapor-intrusion source term has been reduced to a risk-based screening endpoint for every compound present. Because no soil-gas or indoor-air measurements were collected (Section 4.5), no site-specific attenuation factor could be derived and no quantitative indoor-air exposure estimate is presented; the reduction is expressed as a reduction in the soil source term rather than as a modelled change in occupant exposure.

Beyond regulatory compliance, the post-treatment soil concentrations correspond to a substantial reduction in the subsurface source term available to the vapor-intrusion pathway. Pre-treatment xylene concentrations reached 157 mg kg^−1^ at Sector 2 (P6) and 329 mg kg^−1^ in the source zone (P22), with ethylbenzene up to 73.9 mg kg^−1^ at the same borehole—all above Criterion C (50 mg kg^−1^). Ethylbenzene at P22 exceeded its residential-soil screening level (5.8 mg kg^−1^) by more than an order of magnitude, whereas the pre-treatment xylene concentrations, although far above the provincial criterion, remained below the corresponding screening level of 580 mg kg^−1^ [49], a further illustration of the divergence between regulatory and risk-based benchmarks discussed above. Post-treatment, xylenes fell to ≤0.3 mg kg^−1^ and ethylbenzene to ≤0.2 mg kg^−1^ at Sectors 2 and 3, whereas Sector 1 retained a sector mean of 3.5 mg kg^−1^ xylenes (maximum 9.0 mg kg^−1^ at FS9A-E2, above Criterion B) and 1.6 mg kg^−1^ ethylbenzene (maximum 4.4 mg kg^−1^, 76% of its screening level).

Naphthalene, the dominant PAH at this site, is classified as possibly carcinogenic to humans (IARC 2B) [55] and is the priority PAH for vapor intrusion. Post-treatment naphthalene was 0.6 ± 0.3 mg kg^−1^ at Sector 1 and <0.1 mg kg^−1^ at Sector 2 and Sector 3—well below the RPRT residential Criterion B (5 mg kg^−1^). The 77 to >97% naphthalene abatement is therefore directly meaningful from a human-health perspective in addition to satisfying regulatory thresholds.

### 4.3. Surfactant Fate and Residual Ecotoxicity

A key concern when introducing chemical reagents into the subsurface beneath a residence is the toxicological footprint of the reagent itself. Polysorbate-family non-ionic surfactants have been extensively studied for their environmental fate and remediation performance: they are mineralized by non-specialized soil bacteria under the aerobic conditions typical of sandy aquifers, with a reported first-order half-life of 35 ± 2 d in soil microcosms and no persistent transformation products of concern [22,44,47]. The two glycol-ether co-solvents are similarly readily biodegradable, and the inorganic phosphate base and chloride electrolyte introduce no persistent toxicants. Consistent with this biodegradability, the treated effluent met every chemical-quality limit of the municipal discharge permit without any activated-carbon polishing stage (Section 3.4), and the water-based nature of the washing solution eliminated on-site solvent-waste management obligations—a significant practical advantage in an urban residential setting. Low acute toxicity should nevertheless not be equated with ecological innocuity. Acute effects on plankton growth and survival for polysorbate-family surfactants are reported only at concentrations of the order of 100 mg L^−1^, but the same work identifies sublethal effects on grazer physiology at 10 mg L^−1^ and community-level responses at concentrations one to two orders of magnitude lower still [46]. Residual surfactant reaching surface waters therefore remains a legitimate design consideration for surfactant-enhanced remediation even where the formulation is readily biodegradable.

Surfactant concentrations were not measured in extracted water, soil, or groundwater, so residual surfactant cannot be quantified directly. Two components of the washing solution nevertheless acted as field tracers: the chloride electrolyte raises EC and salinity, and the phosphate base raises pH. During the March 2025 injection–diffusion test, salinity reached 7.9–12.6 PSU at 1.0 m from the injection well, against 0.8–0.9 PSU at 2.0 m, and pH reached 10.4–12.6 within 1.0 m. During the final treatment cycle (17–23 November 2025), EC in treatment wells returned to values close to that of the municipal rinse water (0.23 mS cm^−1^) within hours of rinse sequences but reached up to 6.6 mS cm^−1^ after injections, and foam was observed in most wells after injections. The last field record, taken five days before shutdown, still showed EC up to 3.3 mS cm^−1^ and foam in four of the wells sampled. Residual washing solution in the formation at the end of the campaign therefore cannot be excluded, and its fate rests on the biodegradation evidence cited above. EC and pH are already part of the post-treatment groundwater monitoring program.

### 4.4. Implications for Remediation Beneath Occupied Structures

The scarcity of field-scale evidence for in situ remediation beneath occupied structures, noted in Section 1, defines the contribution of the present study. Compared with the two prior campaigns conducted under operational buildings [28,29], this work adds a non-thermal, non-solvent alternative and reports the operational parameters needed to reproduce it: blower capacity, maximum authorized discharge flow, injected volumes expressed as pore volumes of the treated interval, statistical compliance testing, and toxicological screening context.

Vapor migration into the building was managed by engineering control rather than by monitoring: the dual-action well network was operated with extraction continuously exceeding injection, so that induced gradients were directed toward the extraction wells throughout the campaign. The same principle—maintaining active extraction to prevent vapor migration into the occupied structure—was applied by McGee [28] in the only comparable published campaign beneath an occupied apartment building.

### 4.5. Limitations

Several limitations should be acknowledged. (i) The hydrogeological model used a spatially uniform *K* (4.24 × 10^−5^ m s^−1^), the arithmetic mean of five Lefranc tests; subsurface *K* is heterogeneous and a single value may mask preferential flow pathways. (ii) The FEFLOW model did not include reactive transport or biodegradation; aerobic and anaerobic biodegradation of BTEX may have contributed to overall mass removal alongside SER. (iii) A contaminant mass balance could not be closed. The initial inventory could not be estimated reliably, because characterization samples were preferentially collected at the most contaminated horizons and soil bulk density was not measured. Nor could the recovered mass be estimated: untreated extracted water was analyzed only on a few occasions (Section 2.6), the effluent series characterizes the discharge after aeration rather than the extracted mass (Section 3.4), the extracted volume was compiled from operating records rather than metered (Section 2.7), and removal in the soil-gas phase by vacuum extraction, as well as in situ biodegradation, was not quantified. Redistribution of part of the mobilized mass within the subsurface therefore cannot be excluded on mass-balance grounds; the evidence against it is hydraulic (Section 2.7). A flow totalizer, time-resolved analysis of untreated extracted water, and off-gas monitoring are recommended for future campaigns. (iv) Statistical compliance was tested at *n* = 3 discrete samples (Sector 2) and rests on a single sample with its field duplicate at Sector 3; inference at these sample sizes is indicative rather than confirmatory, and the corresponding statistical power is low. (v) The proof-of-concept column experiments used artificially contaminated washed sand, not intact site cores; aged contamination dynamics may differ. (vi) This is a single field-scale case study; broader generalizability requires replication across more complex stratigraphies, higher initial contamination levels, and different NAPL compositions. (vii) Validation samples were preferentially selected on the basis of the highest PID response within each borehole; this purposive sampling design biases the validation dataset toward the most contaminated horizons within each profile. While this makes the compliance demonstration conservative—Criterion B is met even at the worst-case horizons—it limits statistical inference about the average residual concentration across the full treated soil volume. (viii) Contaminant trends were tracked in extracted water rather than in discrete in-well groundwater samples; a well-by-well groundwater time series would better resolve spatial mobilization patterns. (ix) The cost-effectiveness screening criterion was applied relative to internal commercial benchmarks that cannot be disclosed; no absolute cost data are reported, which limits direct economic comparison with alternative technologies. (x) The most contaminated characterization points (P8, P22, P24, P26, P27) could not be re-drilled post-treatment because of physical access constraints (occupied dwelling unit, property-line setback, mature trees). Compliance at those specific points is therefore not directly demonstrated, and the validation dataset should be read as sector-representative rather than exhaustive. Post-treatment monitoring over the planned three-year period is intended to address this residual uncertainty. (xi) No indoor-air or sub-slab soil-gas monitoring was performed during the campaign. Surfactant flushing transiently mobilizes BTEX, and the possibility of a short-term increase in vapor flux toward the overlying dwelling cannot be excluded on the basis of the data collected. Sub-slab and indoor-air monitoring before, during, and after injection should be considered a standard requirement for surfactant-enhanced remediation beneath occupied structures, and its absence here is the principal limitation of the occupied-building component of this study. (xii) Criterion B was not attained at all Sector 1 validation points: total xylenes reached 9.0 mg kg^−1^ at FS9A-E2 against a criterion of 5 mg kg^−1^, and the one-sided 95% UCL of the sector mean (9.4 mg kg^−1^) is likewise above the criterion. A targeted complementary treatment of this residual zone is scheduled for 2026. The performance reported here is therefore that of a single SER cycle, not of a completed remediation at every point of the treated area. (xiii) The soil characterization was designed for contaminant delineation and remediation design rather than for pedological description: pH, organic carbon, cation exchange capacity, bulk density, porosity, specific surface area, and water repellency of the treated interval were not measured, grain size was determined on a single composite sample, and groundwater dissolved oxygen was not recorded. Their influence on sorption and surfactant performance is therefore discussed qualitatively (Section 4.1).

## 5. Conclusions

A full-scale SER campaign was successfully completed beneath an occupied residential building in Quebec City, achieving regulatory compliance with Quebec generic Criterion B at Sectors 2 and 3, with a single residual xylenes exceedance identified at Sector 1 and a substantial reduction in toxicological exposure risk. A network of 46 dual-action injection–extraction wells, installed by direct-push drilling without excavation, delivered 15.3 m^3^ of biodegradable polysorbate-family washing solution and 31.2 m^3^ of water rinse via a stop-and-go protocol over 115 days. Every post-treatment TPH C_10_–C_50_ result at the two sectors where the parameter was analyzed was below Criterion B (700 mg kg^−1^), the highest individual value reaching 416 mg kg^−1^ (59% of the criterion); sector means were 217 mg kg^−1^ (FS10, one-sided 95% UCL 509 mg kg^−1^) and 287 mg kg^−1^ (FS11, single sample and field duplicate). BTEX abatement reached >99% at Sector 2 and naphthalene abatement up to >97%, bringing residual benzene, toluene, xylene, and naphthalene concentrations below the USEPA residential screening levels applicable to the vapor-intrusion pathway. Ethylbenzene remained within 76% of its screening level at the least favorable sample, and 1-methylnaphthalene, although compliant with the provincial criterion, remained 2.2–5.0 times its inhalation-driven screening level at the least favorable sample of each sector—1.7–2.6 times on a sector-mean basis—after a 53–86% reduction. Treated effluent met the City of Quebec municipal sewer discharge permit—including the 6.94 L min^−1^ flow cap—throughout the campaign. The combination of a biodegradable surfactant formulation, absence of excavation, rapid treatment duration, and explicit toxicological framing positions this approach as a viable, low-impact option for petroleum remediation at urban confined sites. As part of standard practice, a structured post-treatment monitoring program of at least three years will confirm long-term stability and detect any contaminant rebound.

## Figures and Tables

**Figure 2:**
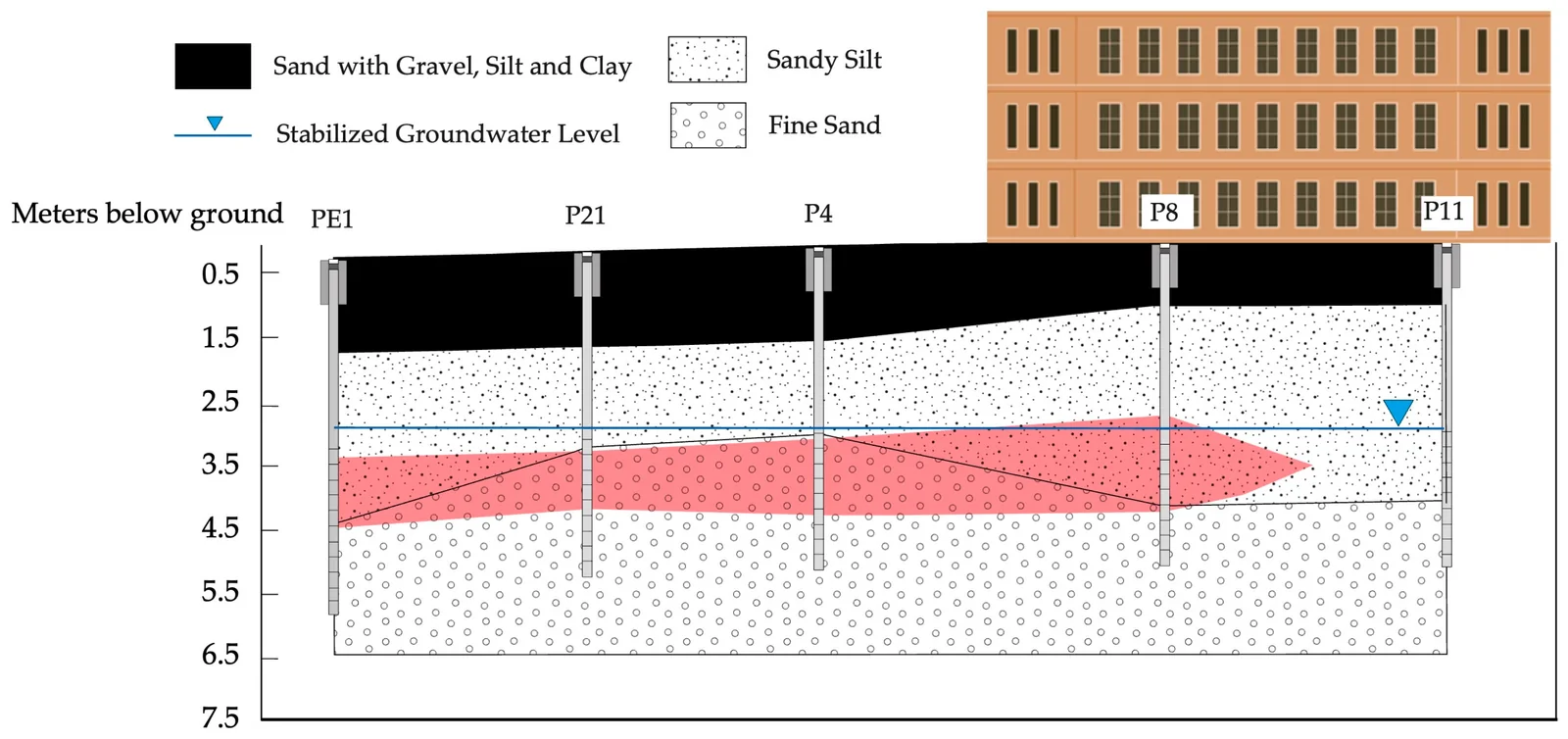
Conceptual hydrogeological cross-section through the source zone along trace A–A′ (Figure 1), extending from the exterior lawn area (PE1) beneath the occupied residential building (P8, P11). Vertical axis: depth below ground surface (m). The stratigraphic sequence comprises a surficial sandy silt, an underlying fine sand hosting the water table, and a basal sand with gravel, silt, and clay; composite grain-size analysis classifies the treated interval as a fine-to-medium sand (93.7% sand, 6.0% silt + clay, 0.3% gravel; Section 3.1). Blue line: stabilized groundwater level (≈2.9 m bgs), measured in six screened wells and corrected for tidal and barometric variation (Section 2.2.1). Red envelope: interpreted extent of the PHC-impacted interval (3.0–4.5 m bgs), reconstructed from continuous soil cores, organoleptic indicators (visual staining, petrochemical odor), and PID headspace screening; this interval corresponds to the capillary fringe and upper saturated zone targeted by the 1.5 m screened sections of the treatment wells. Vertical scale as indicated; horizontal well spacing is schematic and not to scale. Well casings are shown diagrammatically.

**Figure 3:**
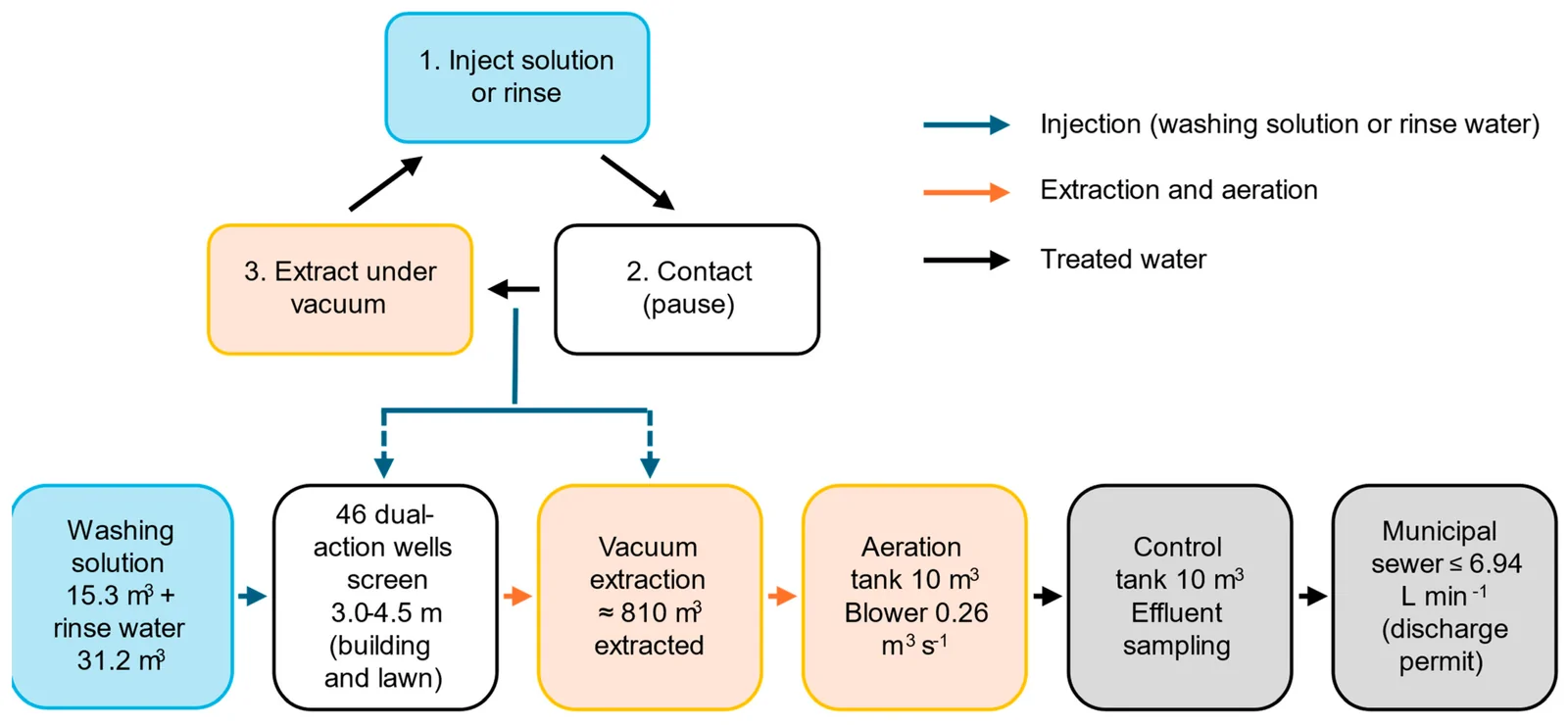
Schematic of the treatment system. Washing solution (15.3 m^3^) and rinse water (31.2 m^3^) were injected at low pressure into 46 dual-action wells screened over 3.0–4.5 m bgs, beneath the building and in the lawn area, following a stop-and-go cycle (injection, contact period, extraction under vacuum). Extracted water (≈810 m^3^) was treated by diffused-air aeration in a 10 m^3^ tank, checked in a 10 m^3^ control tank (effluent sampling, 10 events), and discharged to the municipal combined sewer at ≤6.94 L min^−1^.

**Figure 4:**
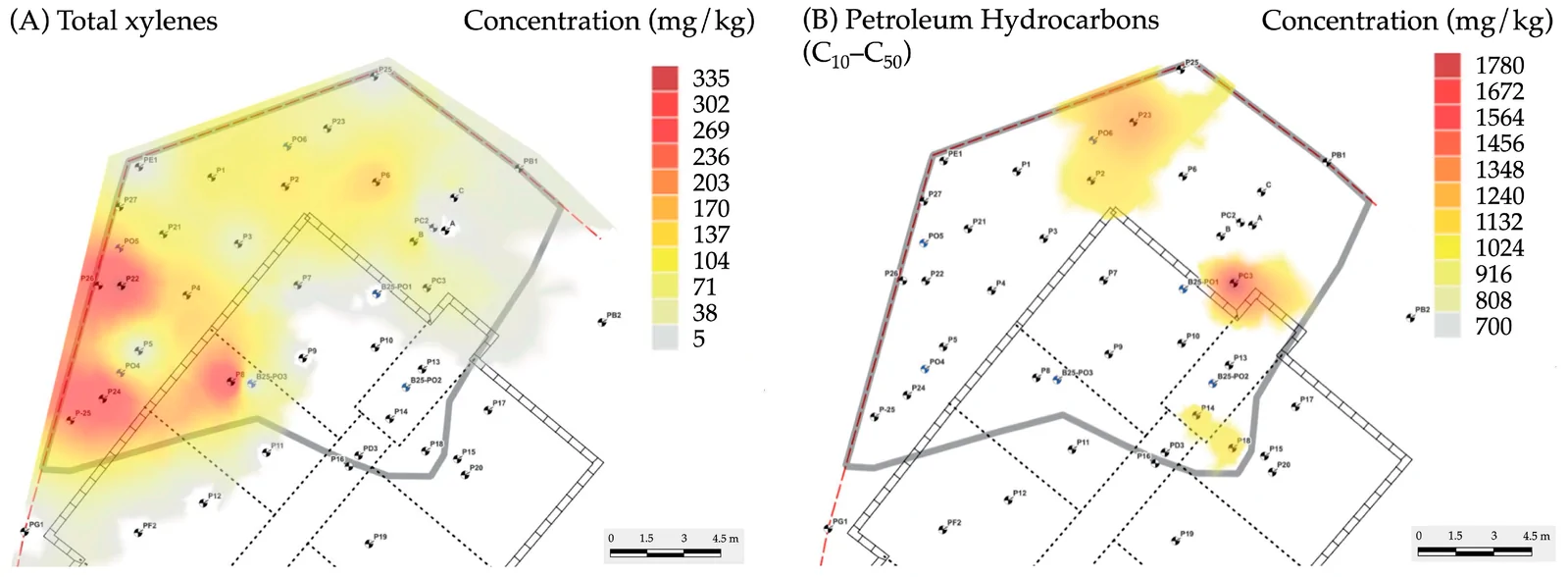
Kriged soil-concentration maps of the characterization dataset (Table S1; 43 samples, 6 of which were collected during the active remediation phase) for (**A**) total xylenes and (**B**) C_10_–C_50_ petroleum hydrocarbons. The lower bound of each color scale is set at the corresponding Quebec MELCCFP Criterion B threshold (RPRT, residential use): 5 and 700 mg kg^−1^, respectively; any colored area therefore exceeds the regulatory limit. Warm colors denote increasing concentration. Black circles: delineation boreholes and injection–extraction wells (P-series); blue circles: monitoring wells (PO- series). Grey outline: area of interest; hatched line: footprint of the residential building; dotted lines: internal partition walls; red dashed line: property boundary. Scale bar: 4.5 m. Interpolated surfaces are illustrative only; measured values are given in Table S1.

**Figure 5:**
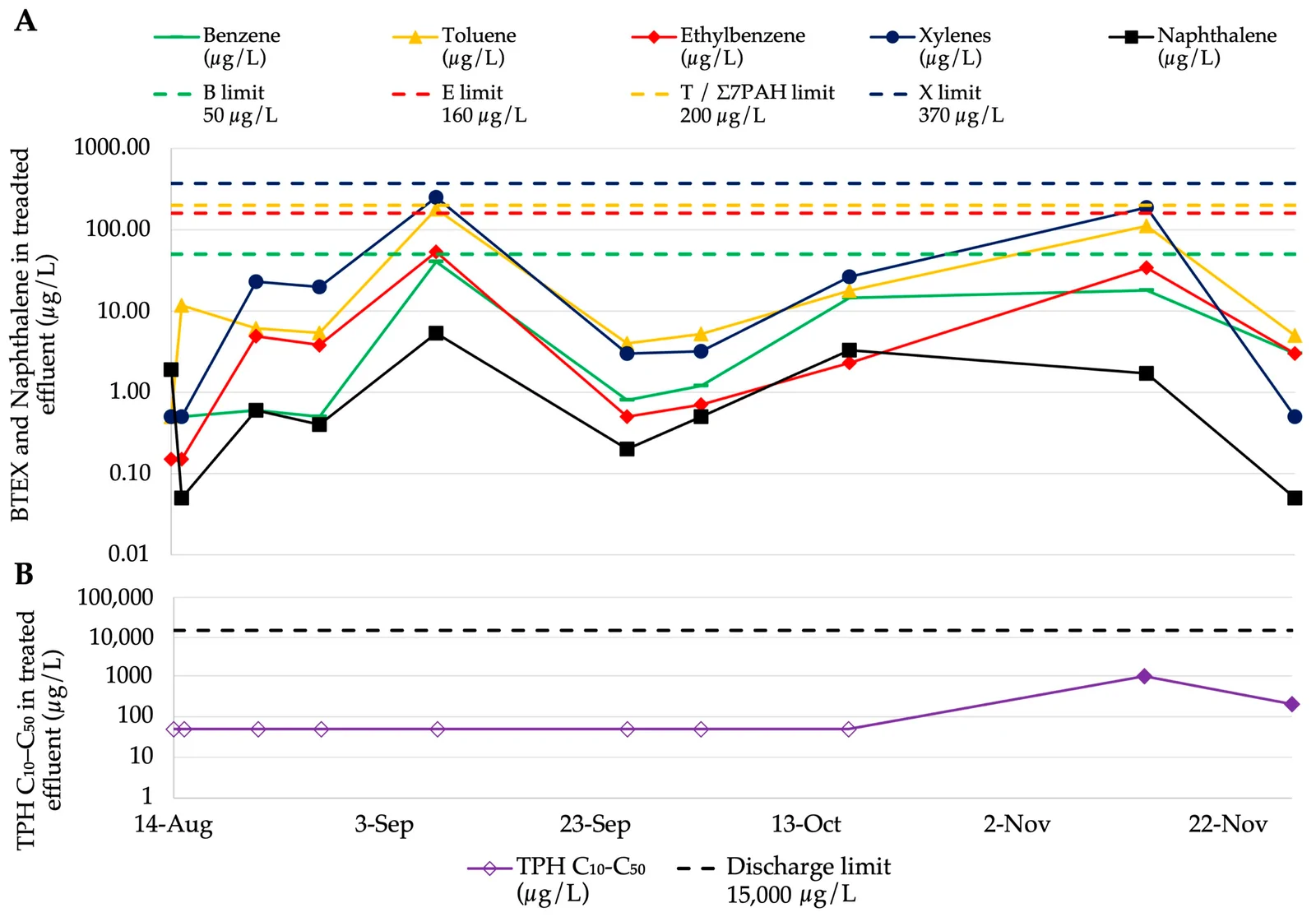
Time series of treated effluent quality during the SER campaign (14 August–28 November 2025), monitored over 10 discrete sampling events. (**A**) BTEX compounds and naphthalene (log scale): benzene (green), toluene (yellow), ethylbenzene (red), xylenes (blue), naphthalene (black). (**B**) Total petroleum hydrocarbons C_10_–C_50_ (purple); open symbols indicate values < LOQ, filled symbols indicate detected concentrations. Dashed horizontal lines: maximum allowable discharge concentrations to the municipal sewer: B = 50 µg L^−1^, T = 200 µg L^−1^, E = 160 µg L^−1^, X = 370 µg L^−1^, naphthalene included in ΣPAH = 200 µg L^−1^; TPH C_10_–C_50_ = 15,000 µg L^−1^. All measured values remained below their respective regulatory thresholds throughout the campaign.

**Table 1:** Pre- and post-treatment contaminant concentrations and abatement at the three validation sectors (3.0–4.5 m bgs).

Parameter	Unit	Crit. B ^1^	Pre P3 (S1) ^3^	Post FS9 (S1)	Pre P6 (S2) ^3^	Post FS10 (S2)	Pre P14 (S3) ^3^	Post FS11 (S3)
TPH C_10_–C_50_	mg kg^−1^	700	226	n.a. ^2^	544	217 ± 173	741	381; 192
Naphthalene	mg kg^−1^	5	2.8	0.6 ± 0.3	4.2	<0.1	1.2	<0.1
1-Methylnaphthalene	mg kg^−1^	1	1.0	0.5 ± 0.2	1.7	<0.1–0.9	2.2	0.4; 0.2
2-Methylnaphthalene	mg kg^−1^	1	2.8	0.5 ± 0.1	4.1	<0.1–0.4	4.6	0.3; 0.2
Ethylbenzene	mg kg^−1^	5	7.4	1.6 ± 1.8	35.8	<0.2	0.8	n.a. ^2^
Xylenes	mg kg^−1^	5	36.1	3.5 ± 3.5	157.0	<0.2–0.3	1.3	n.a. ^2^
Benzene	mg kg^−1^	0.5	<0.1	<0.1	<0.1	<0.1	<0.1	n.a. ^2^
Toluene	mg kg^−1^	3	<0.2	<0.2	0.3	<0.2	<0.2	n.a. ^2^

^1^ Quebec generic Criterion B for residential use [10]. ^2^ n.a. = not analyzed post-treatment. Values are mean ± SD for *n* = 3 discrete samples (FS9, FS10) and individual values for FS11 (one sample and its field duplicate). Laboratory duplicate and triplicate determinations are averaged to the sample level and are not counted as independent observations. ^3^ Pre-treatment values are those of a single reference borehole per sector: P3 (3.31–3.39 m bgs) for Sector 1, P6 (3.15–3.65 m) for Sector 2, and P14 (3.60–3.85 m) for Sector 3 (Table S1).

## Data Availability

All analytical data generated or analyzed in this study are included in this article and in the Supplementary Materials (Tables S1–S8). Laboratory certificates of analysis are available from the corresponding author on reasonable request. The composition of the washing solution is subject to a confidentiality agreement and is described in Section 2.9 at the level of chemical family and working concentration.

## Supplementary Materials

The following supporting information can be downloaded at: https://www.mdpi.com/article/10.3390/toxics14100906/s1, Table S1: pre-remediation and in-SER soil analytical results; Table S2: post-treatment validation results by sector; Table S3: per-sample validation results, all sectors; Table S4: field demonstration test validation results (FV1–FV4); Table S5: treated effluent monitoring; Table S6: fluid delivery log and pore-volume normalization; Table S7: soil and groundwater properties; Table S8: Quebec generic soil criteria and CCME soil quality guidelines.

## Abbreviations

The following abbreviations are used in this manuscript:

bgs	Below ground surface
BNQ	Bureau de normalisation du Québec
BTEX	Benzene, toluene, ethylbenzene and xylenes
CCME	Canadian Council of Ministers of the Environment
CEAEQ	Centre d’expertise en analyse environnementale du Québec
CFM	Cubic feet per minute
CMC	Critical micelle concentration
DNAPL	Dense non-aqueous phase liquid
EC	Electrical conductivity
EC_50_	Median effective concentration
FEFLOW	Finite Element subsurface FLOW system
GC–FID	Gas chromatography—flame ionization detection
GC–MS	Gas chromatography—mass spectrometry
HP C_10_–C_50_	Hydrocarbures pétroliers C_10_–C_50_ (Quebec regulatory term for TPH C_10_–C_50_)
IARC	International Agency for Research on Cancer
K	Saturated hydraulic conductivity
LNAPL	Light non-aqueous phase liquid
LOD	Limit of detection
MDDEP	Ministère du Développement durable, de l’Environnement et des Parcs (Quebec)
MELCCFP	Ministère de l’Environnement, de la Lutte contre les changements climatiques, de la Faune et des Parcs (Quebec)
NAPL	Non-aqueous phase liquid
ORP	Oxidation–reduction potential
PAH	Polycyclic aromatic hydrocarbon
PCB	Polychlorinated biphenyl
PHC	Petroleum hydrocarbon
PID	Photoionization detector
PSI	Pounds per square inch
PSU	Practical salinity unit
PV	Pore volume
QA/QC	Quality assurance/quality control
RPRT	Règlement sur la protection et la réhabilitation des terrains (Quebec)
RSL	Regional screening level (USEPA)
SD	Standard deviation
SEAR	Surfactant-enhanced aquifer remediation
SER	Surfactant-enhanced remediation
SIM	Selected ion monitoring
TPH	Total petroleum hydrocarbons
UCL	Upper confidence limit
USDA	United States Department of Agriculture
USEPA	United States Environmental Protection Agency
UST	Underground storage tank
VOC	Volatile organic compound
