# Supplementary material for: Surfactant-Enhanced In Situ Remediation Beneath an Occupied Residential Building: Reduction of the BTEX Vapor-Intrusion Source Term and Field Validation in a Single Field Season
Source: Toxics. 2026 Oct 14;14(10):906. doi: 10.3390/toxics14100906 (PMC13709739; doi:10.3390/toxics14100906)
Supplement: Supplementary file 1 [file toxics-14-00906-s001.zip › toxics-4576152-supplementary.pdf]

Supplementary Materials

Surfactant-Enhanced In Situ Remediation Beneath an Occupied Residential Building: Reduction of the BTEX Vapor-Intrusion Source Term and Field Validation in a Single Field Season

Gautier Decabooter — Biotix Inc., Quebec City, QC, Canada. Correspondence: gdecabooter@biotix.ca

Contents: Table S1 — pre-remediation and in-SER soil analytical results; Table S2 — post-treatment validation results by sector; Table S3 — per-sample validation results, all sectors; Table S4 — field demonstration test validation results (FV1–FV4); Table S5 — treated effluent monitoring; Table S6 — fluid delivery log and pore-volume normalization; Table S7 — soil and groundwater properties; Table S8 — Quebec generic soil criteria compared with CCME soil quality guidelines.

General notes. All soil concentrations are reported in mg kg^{-1} dry weight and all water concentrations in µg L^{-1}, as issued by the accredited external laboratory (Section 2.10). Values preceded by "<" are below the reported detection limit (RDL). Where summary statistics are computed over datasets containing non-detects, non-detects are substituted at the full RDL, which yields a conservative (upper-bound) estimate of the residual concentration; the alternative half-RDL substitution is reported in the footnotes where it changes a stated value. Laboratory duplicate (DUP) and triplicate (TRIP) determinations are replicate analyses of a single soil sample and are averaged to the sample level before any statistic is computed; they are not treated as independent observations. Quebec generic criteria A/B/C for residential land use are those of the MELCCFP Guide d'intervention (2021) and Annexes I and II of the RPRT. bgs = below ground surface. Cells shaded pink denote a result above Quebec Criterion B; cells shaded amber denote a residual hot spot below Criterion B. Reference numbers refer to the reference list of the main text.

Table S1. Pre-remediation and in-SER soil analytical results

Soil analytical results for all non-validation boreholes across four campaigns. The full-scale remediation campaign ran from 12 August to 5 December 2025; samples labeled MON were collected during the active remediation phase, not after it. TPH C_{10}–C_{50} = total petroleum hydrocarbons in the C_{10}–C_{50} range, designated hydrocarbures pétroliers (HP C_{10}–C_{50}) in the Quebec regulatory framework. The reporting limit is 0.1 mg kg^{-1} for naphthalene and for each of the methylnaphthalene and dimethylnaphthalene isomers. ND = not analyzed; <100 = below the TPH reporting threshold of 100 mg kg^{-1}.

Borehole	Date (dd-mm-yyyy)	Depth (m bgs)	Camp.[b]	Class[c]	TPH C_{10}–C_{50}	Naphthalene	1-Methyl-naphth.	2-Methyl-naphth.	1,3-DM-naphth.	Benzene	Toluene	Ethyl-benzene	Xylenes
					(mg kg^{-1})	(mg kg^{-1})	(mg kg^{-1})	(mg kg^{-1})	(mg kg^{-1})	(mg kg^{-1})	(mg kg^{-1})	(mg kg^{-1})	(mg kg^{-1})
PB1	12-09-2024	2.70–3.00	PIII	B–C	468	3.0	2.4	4.4	0.6	0.1	0.2	3.3	13.1
PB2	12-09-2024	4.50–5.00	PIII	≤A	<100	<0.1	<0.1	<0.1	<0.1	0.1	0.2	0.2	0.2
PC3	12-09-2024	4.00–4.50	PIII	>C	1780	9.7	5.0	11.4	2.3	0.1	1.3	13.2	48
PE1	23-09-2024	3.08–3.38	PIII	B–C [a]	267	0.6	2.4	1.6	1.7	ND	ND	ND	ND
PF2	23-09-2024	3.60–4.05	PIII	≤A [a]	<100	<0.1	<0.1	<0.1	<0.1	ND	ND	ND	ND
PG1	23-09-2024	3.25–3.75	PIII	≤A [a]	<100	<0.1	<0.1	<0.1	<0.1	ND	ND	ND	ND
PD3	24-09-2024	3.60–4.10	PIII	B–C [a]	271	0.5	0.7	1.6	0.2	ND	ND	ND	ND
PF3	24-09-2024	3.55–4.05	PIII	≤A [a]	<100	<0.1	<0.1	<0.1	<0.1	ND	ND	ND	ND
P8	25-03-2025	2.70–3.20	INT	>C	518	5.7	1.6	4.2	0.7	<0.1	8.6	20.1	124
P8	25-03-2025	3.60–4.10	INT	>C	333	3.6	1.0	2.7	0.4	0.6	48.5	60.9	335
P9	20-03-2025	3.60–3.95	INT	A–B	130	<0.1	<0.1	<0.1	<0.1	<0.1	<0.2	<0.2	1.0
P10	20-03-2025	3.72–4.11	INT	A–B	217	0.2	0.4	0.2	0.1	<0.1	<0.2	0.8	1.3
P11	25-03-2025	3.60–4.10	INT	A–B	<100	<0.1	<0.1	<0.1	<0.1	<0.1	<0.2	<0.2	0.5
P12	19-03-2025	3.60–4.10	INT	≤A	<100	<0.1	<0.1	<0.1	<0.1	<0.1	<0.2	<0.2	<0.2
P13	25-03-2025	3.85–4.15	INT	A–B	<100	0.2	0.2	0.2	<0.1	<0.1	<0.2	0.4	0.4

Borehole	Date (dd-mm-yyyy)	Depth (m bgs)	Camp.[b]	Class[c]	TPH C_{10}–C_{50}	Naphthalene	1-Methyl-naphth.	2-Methyl-naphth.	1,3-DM-naphth.	Benzene	Toluene	Ethyl-benzene	Xylenes
					(mg kg^{-1})	(mg kg^{-1})	(mg kg^{-1})	(mg kg^{-1})	(mg kg^{-1})	(mg kg^{-1})	(mg kg^{-1})	(mg kg^{-1})	(mg kg^{-1})
P14	25-03-2025	3.60–3.85	INT	B–C	741	1.2	2.2	4.6	0.9	<0.1	<0.2	0.8	1.3
P15	25-03-2025	3.78–4.20	INT	A–B	100	0.3	0.3	0.8	<0.1	<0.1	<0.2	<0.2	0.2
P16	26-03-2025	3.60–3.95	INT	≤A	<100	<0.1	<0.1	<0.1	<0.1	<0.1	<0.2	<0.2	<0.2
P17	19-03-2025	3.60–3.97	INT	≤A	<100	<0.1	<0.1	<0.1	<0.1	<0.1	<0.2	<0.2	<0.2
P18	19-03-2025	3.60–3.82	INT	B–C	775	0.4	1.5	1.2	0.6	<0.1	<0.2	<0.2	0.2
P19	19-03-2025	3.60–3.80	INT	≤A	<100	<0.1	<0.1	<0.1	<0.1	<0.1	<0.2	<0.2	<0.2
P20	26-03-2025	3.60–4.10	INT	≤A	<100	<0.1	<0.1	<0.1	<0.1	<0.1	<0.2	<0.2	<0.2
B25-PO1	26-03-2025	3.80–4.10	INT	A–B	227	0.5	0.5	1.0	0.3	<0.1	<0.2	0.5	1.8
B25-PO2	26-03-2025	3.69–3.95	INT	A–B [a]	<100	0.2	0.3	0.7	0.1	ND	ND	ND	ND
B25-PO3	26-03-2025	3.60–4.10	INT	B–C	<100	0.6	0.2	0.5	0.1	<0.1	<0.2	1.4	6.4
P1	29-04-2025	3.75–3.95	EXT	>C	177	3.0	0.7	1.9	0.3	0.5	2.1	34.3	103
P2	28-04-2025	3.43–3.87	EXT	>C	778	10.8	2.9	7.9	0.8	<0.1	0.4	26.7	130
P3	29-04-2025	3.00–3.23	EXT	B–C	351	0.6	1.6	0.2	0.2	<0.1	<0.2	3.6	11.9
P3	29-04-2025	3.31–3.39	EXT	B–C	226	2.8	1.0	2.8	0.2	<0.1	<0.2	7.4	36.1
P4	28-04-2025	3.33–3.52	EXT	>C	405	5.2	1.9	4.8	0.4	<0.1	1.5	58.6	146
P5	28-04-2025	3.40–3.90	EXT	B–C	<100	0.5	0.1	0.3	<0.1	<0.1	0.6	3.5	18.4
P6	29-04-2025	3.15–3.65	EXT	>C	544	4.2	1.7	4.1	0.5	<0.1	0.3	35.8	157
P21	29-04-2025	3.10–3.60	EXT	>C	127	1.6	0.6	1.5	0.2	0.1	4.2	23.9	91.5
P22	28-04-2025	3.00–3.25	EXT	>C	216	4.2	1.0	2.2	0.5	0.5	20.6	73.9	329
P23	29-04-2025	3.09–3.57	EXT	>C	1140	10.7	3.9	6.8	0.8	<0.1	<0.2	21.2	57.8
P24	28-04-2025	3.10–3.25	EXT	>C	653	7.6	2.1	5.1	1.0	0.4	17.5	66.9	305
P25	29-04-2025	3.00–3.20	EXT	B–C	540	2.3	2.2	2.7	0.4	<0.1	<0.2	3.2	7.3
PO4	09-09-2025	4.00–4.20	MON	B–C	<100	0.3	0.2	0.3	0.1	<0.1	0.2	3.5	17.7
PO5	09-09-2025	2.50–2.65	MON	B–C	160	2.4	1.1	2.0	1.0	<0.1	0.2	3.3	12.7
PO6	09-09-2025	3.20–3.70	MON	>C	399	2.5	2.4	5.6	0.8	<0.1	<0.2	14.9	77.2
P26	22-10-2025	3.00–3.30	MON	>C	201	2.8	0.7	1.8	0.5	0.1	4.5	38.8	230
P27	22-10-2025	3.40–3.75	MON	>C	148	3.0	0.9	2.1	0.5	0.8	26.2	47.2	259
P25 (mon.)	22-10-2025	3.40–3.75	MON	≤A	<100	<0.1	<0.1	<0.1	0.1	<0.1	<0.2	<0.2	<0.2

[a]BTEX not analyzed at this borehole; the class is based on TPH C_{10}–C_{50}, naphthalene and the methylnaphthalene and dimethylnaphthalene isomers only.

[b]Campaign codes: PIII = Phase III delineation (Sep–Oct 2024); INT = SER well installation, interior boreholes drilled through the building slab (March–April 2025); EXT = SER well installation, exterior boreholes in lawn/garden areas (April 2025); MON = in-SER monitoring (Sep and Oct 2025).

[c]Class = the most restrictive classification obtained across all analyzed parameters. Criteria A/B/C (mg kg^{-1}): TPH C_{10}–C_{50} 100/700/3,500; naphthalene 0.1/5/50; 1-methylnaphthalene, 2-methylnaphthalene and 1,3-dimethylnaphthalene 0.1/1/10 each; benzene 0.2/0.5/5; toluene 0.2/3/30; ethylbenzene 0.2/5/50; xylenes 0.4/5/50. Classes are reported as intervals: ≤A, A–B (> A and ≤ B), B–C (> B and ≤ C), >C (above Criterion C). Shaded rows denote samples above Criterion C.

Summary of the 43 samples: 10 ≤A, 7 A–B, 12 B–C and 14 >C. Thirteen of the >C classifications were driven by BTEX — principally xylenes (13 samples, maximum 335 mg kg^{-1} at P8) and ethylbenzene (4 samples, maximum 73.9 mg kg^{-1} at P22), with toluene at one sample (48.5 mg kg^{-1} at P8) — and one by 2-methylnaphthalene (PC3, 11.4 mg kg^{-1} against a Criterion C of 10 mg kg^{-1}). No sample exceeded Criterion C for TPH C_{10}–C_{50}; five exceeded Criterion B (maximum 1,780 mg kg^{-1} at PC3). Naphthalene exceeded Criterion B at six samples (maximum 10.8 mg kg^{-1} at P2) and benzene at two (P8, 0.6; P27, 0.8 mg kg^{-1}). The methylnaphthalene isomers, each subject to a Criterion B of 1 mg kg^{-1}, were the most

frequently exceeded parameter group at the site: 23 samples exceeded Criterion B for at least one of 1-methylnaphthalene (15 samples), 2-methylnaphthalene (22 samples) and 1,3-dimethylnaphthalene (2 samples), with maxima of 5.0, 11.4 and 2.3 mg kg^{-1} respectively, all at PC3.

Table S2. Post-treatment validation results by sector

Validation sampling (5 December 2025) at the three contamination sectors. One PID-selected soil sample was submitted per validation borehole: three boreholes at Sector 1 (FS9A, FS9B, FS9C), three at Sector 2 (FS10A, FS10B, FS10C) and one at Sector 3 (FS11). Samples were preferentially selected at the horizon of highest PID response within each profile; the dataset is therefore biased toward the most contaminated horizons, which makes the compliance demonstration conservative. Values are mean ± SD where n = 3, ranges where individual results are more informative than a mean, and individual values at Sector 3. Per-sample results including all replicate determinations are given in Table S3.

Sector / validation borehole	n	TPH C_{10}–C_{50}	Naphtha-lene	1-Methyl-naphthalene	2-Methyl-naphthalene	1,3-Dimethyl-naphthalene	Ethyl-benzene	Xylenes
		(mg kg^{-1})	(mg kg^{-1})	(mg kg^{-1})	(mg kg^{-1})	(mg kg^{-1})	(mg kg^{-1})	(mg kg^{-1})
Sector 1 — FS9 (110 m²)	3	n.a.d	0.6 ± 0.3	0.5 ± 0.2	0.5 ± 0.1	≤ 0.1	1.6 ± 1.8	3.5 ± 3.5 e
Sector 2 — FS10 (100 m²)	3	217 ± 173 f	<0.1	<0.1–0.9	<0.1–0.4	<0.1–0.5	<0.2–0.2	<0.2–0.3
Sector 3 — FS11 (25 m²)	1 + field dup.	381; 192 g	<0.1	0.4; 0.2	0.3; 0.2	0.2; 0.1	n.a.h	n.a.h
**Quebec Criterion B (residential)**	—	**700**	**5**	**1**	**1**	**1**	**5**	**5**

[d]n.a. = not analyzed post-treatment. TPH C_{10}–C_{50} was not analyzed at FS9: pre-treatment concentrations at this sector were already below Criterion B (226–351 mg kg^{-1} at P3, Table S1). Remediation at Sector 1 was driven by ethylbenzene (7.4 mg kg^{-1}) and xylenes (36.1 mg kg^{-1}), both above Criterion B, which were prioritized for post-treatment analysis.

[e]Residual exceedance of Criterion B. Sample FS9A-E2 returned 9.0 mg kg^{-1} total xylenes, confirmed at 6.8 and 6.9 mg kg^{-1} in duplicate and triplicate determinations of the same sample (Table S3); the sector mean of the three samples is 3.5 mg kg^{-1} and the one-sided 95 % UCL of the mean is 9.4 mg kg^{-1}, also above Criterion B. Criterion B was therefore not attained at all Sector 1 validation points. Ethylbenzene at the same sample reached 4.4 mg kg^{-1}, below but close to Criterion B (5 mg kg^{-1}); the sector one-sided 95 % UCL is 4.6 mg kg^{-1}. A targeted complementary treatment of this residual zone is scheduled for 2026 (Section 4.5 of the main text).

[f]Mean ± SD of the three TPH C_{10}–C_{50} analyses at FS10 (134, 416 and < 100 mg kg^{-1}), with the non-detect substituted at the 100 mg kg^{-1} reporting limit. Half-RDL substitution gives 200 ± 192 mg kg^{-1}. The highest individual value was 416 mg kg^{-1}, i.e. 59 % of Criterion B. One-sided 95 % UCL of the mean = 509 mg kg^{-1}; one-sample one-tailed t-test against Criterion B, $t(2) = -4.83$, $p = 0.020$.

[g]Individual values are reported for Sector 3 because validation there rests on a single soil sample (FS11-E4) and its field duplicate. The relative percent difference between the two determinations is 66 %, of the same order as the 55 % recorded during the field demonstration test (Table S4); both results are below Criterion B and no inferential test was performed.

[h]BTEX at FS11 were not analyzed post-treatment: pre-treatment concentrations at P14 were already below Criterion B (ethylbenzene 0.8 mg kg^{-1}; xylenes 1.3 mg kg^{-1}; Criterion B = 5 mg kg^{-1}).

The Quebec Criterion B for the methylnaphthalene and dimethylnaphthalene isomers (1 mg kg^{-1}) applies to each isomer individually. No methylnaphthalene or dimethylnaphthalene result exceeded Criterion B at any validation sample; the highest was 0.9 mg kg^{-1} (1-methylnaphthalene, FS10B-E2).

Table S3. Per-sample validation results, all sectors, 5 December 2025

Individual laboratory results for the seven discrete validation soil samples and all replicate determinations. Seven soil samples were collected across seven closely-spaced validation boreholes grouped in three sectors; BTEX were determined in analytical triplicate on five of these samples and a field duplicate was collected at FS11-E4, giving 16 BTEX, 5 TPH C_{10}–C_{50} and 8 PAH determinations in total. All 16 PAH other than naphthalene, 1-methylnaphthalene, 2-methylnaphthalene, 1,3-dimethylnaphthalene and 2,3,5-trimethylnaphthalene were below the reporting limit at every sample and are omitted for brevity; 2,3,5-trimethylnaphthalene was below the reporting limit at every sample and is likewise omitted.

Sector	Sample ID	Replicate	TPH C_{10}–C_{50}	Chrom. region	Naphtha-lene	1-Methyl-naphth.	2-Methyl-naphth.	1,3-DM-naphth.	B	T	E	X	Moist. (%)
1	FS9A-E2	—	n.a.	—	0.8	0.7	0.4	0.1	<0.1	<0.2	4.4	9.0	14.5
1	FS9A-E2	DUP	—	—	—	—	—	—	<0.1	<0.2	3.1	6.8	14.5
1	FS9A-E2	TRIP	—	—	—	—	—	—	<0.1	<0.2	3.4	6.9	14.5
1	FS9B-E7	—	n.a.	—	0.3	0.3	0.4	<0.1	<0.1	<0.2	0.9	1.9	16.0
1	FS9B-E7	DUP	—	—	—	—	—	—	<0.1	<0.2	0.6	1.3	16.0

Sector	Sample ID	Replicate	TPH C_{10}–C_{50}	Chrom. region	Naphtha-lene	1-Methyl-naphth.	2-Methyl-naphth.	1,3-DM-naphth.	B	T	E	X	Moist. (%)
1	FS9B-E7	TRIP	—	—	—	—	—	—	<0.1	<0.2	0.7	1.5	16.0
1	FS9C-E4	—	n.a.	—	0.8	0.4	0.6	0.1	<0.1	<0.2	<0.2	<0.2	14.4
1	FS9C-E4	DUP	—	—	—	—	—	—	<0.1	<0.2	0.6	2.7	14.4
1	FS9C-E4	TRIP	—	—	—	—	—	—	<0.1	<0.2	0.4	1.3	14.4
2	FS10A-E4	—	134	A-B-C	<0.1	<0.1	<0.1	<0.1	<0.1	<0.2	0.2	0.2	17.7
2	FS10A-E4	DUP	—	—	—	—	—	—	<0.1	<0.2	<0.2	<0.2	17.7
2	FS10A-E4	TRIP	—	—	—	—	—	—	<0.1	<0.2	<0.2	<0.2	17.7
2	FS10B-E2	—	416	A	<0.1	0.9	0.4	0.5	<0.1	<0.2	<0.2	0.3	15.0
2	FS10C-E3	—	<100	NA	<0.1	<0.1	<0.1	<0.1	<0.1	<0.2	<0.2	<0.2	14.9
2	FS10C-E3	DUP	—	—	—	—	—	—	<0.1	<0.2	<0.2	<0.2	14.9
2	FS10C-E3	TRIP	—	—	—	—	—	—	<0.1	<0.2	<0.2	<0.2	14.9
3	FS11-E4	—	381	A	<0.1	0.4	0.3	0.2	n.a.	n.a.	n.a.	n.a.	15.2
3	FS11-E4	Field dup.	192	A	<0.1	0.2	0.2	0.1	n.a.	n.a.	n.a.	n.a.	15.5
Criterion B	(residential)	—	700	—	5	1	1	1	0.5	3	5	5	—

Reporting limits: TPH C_{10}–C_{50} 100; naphthalene, methylnaphthalenes and dimethylnaphthalene 0.1; benzene 0.1; toluene, ethylbenzene and xylenes 0.2 mg kg^{-1}. "—" = parameter not determined on this replicate. n.a. = parameter not analyzed on this sample. Chromatographic region: A = light (gasoline-range), B = middle distillate, C = heavy; NA = not assigned (TPH below the reporting limit).

Surrogate recoveries were within the laboratory acceptance limits throughout (fluorobenzene 78–116 %, limits 50–140 %; naphthalene-d8 83–103 %; pyrene-d10 89–131 %; p-terphenyl-d14 96–132 %; nonane 78–116 %, limits 60–140 %). Moisture content ranged from 14.4 to 17.7 %.

Sample FS9A-E2 (shaded pink) is the residual hot spot of Sector 1: total xylenes 9.0 mg kg^{-1} against a Criterion B of 5 mg kg^{-1}, confirmed at 6.8 and 6.9 mg kg^{-1} in duplicate and triplicate analysis, with ethylbenzene at 4.4 mg kg^{-1} (88 % of Criterion B). Sample FS10B-E2 (shaded amber) is the residual hot spot of Sector 2: it carries the highest TPH C_{10}–C_{50} (416 mg kg^{-1}, 59 % of Criterion B), the only methylnaphthalene and dimethylnaphthalene detections of the sector, and a chromatographic region assignment of A (light, gasoline-range hydrocarbons); all its results are below Criterion B.

Table S4. Field demonstration test — validation results (FV1–FV4)

Post-treatment soil results for the September 2024 single-well field demonstration test, in which approximately 3,600 L of washing solution followed by 3,200 L of water rinse were injected via well PC3 (Section 2.3.2). Validation boreholes FV1–FV4 were drilled on 2 October 2024 and yielded 11 discrete soil samples plus one field duplicate. The pre-treatment reference for this test is borehole PC3 (12 September 2024): TPH C_{10}–C_{50} 1,780; naphthalene 9.7; benzene 0.1; toluene 1.3; ethylbenzene 13.2; xylenes 48 mg kg^{-1} (Table S1).

Sample	TPH C_{10}–C_{50}	Naphtha-lene	1-Methyl-naphth.	2-Methyl-naphth.	B	T	E	X
	(mg kg^{-1})	(mg kg^{-1})	(mg kg^{-1})	(mg kg^{-1})	(mg kg^{-1})	(mg kg^{-1})	(mg kg^{-1})	(mg kg^{-1})
FV1-E6	<100	<0.1	<0.1	<0.1	—	—	—	—
FV1-E7	321	0.3	0.9	0.2	<0.1	<0.2	0.3	0.4
FV1-E7 (field duplicate)	567	0.8	1.6	0.5	—	—	—	—
FV1-E8	306	1.6	1.0	1.7	—	—	—	—
FV1-E10	<100	<0.1	<0.1	<0.1	—	—	—	—
FV1-E11	<100	<0.1	<0.1	<0.1	—	—	—	—
FV2-E7	156	<0.1	<0.1	0.2	<0.1	<0.2	<0.2	0.2
FV2-E8	<100	<0.1	<0.1	<0.1	—	—	—	—
FV2-E10	436	<0.1	<0.1	<0.1	—	—	—	—
FV3-E6	150	<0.1	0.2	0.2	<0.1	<0.2	0.5	0.4
FV3-E8	<100	<0.1	<0.1	<0.1	—	—	—	—
FV4-E6	156	0.4	0.4	0.9	<0.1	<0.2	0.4	0.7

Sample	TPH C_{10}–C_{50}	Naphtha-lene	1-Methyl-naphth.	2-Methyl-naphth.	B	T	E	X
	(mg kg^{-1})	(mg kg^{-1})	(mg kg^{-1})	(mg kg^{-1})	(mg kg^{-1})	(mg kg^{-1})	(mg kg^{-1})	(mg kg^{-1})
Criterion B (residential)	700	5	1	1	0.5	3	5	5

"—" = parameter not determined on this sample. BTEX were determined on one sample per borehole (FV1-E7, FV2-E7, FV3-E6, FV4-E6); TPH C_{10}–C_{50} and PAH were determined on all samples. All 16 PAH other than the naphthalene series were below the reporting limit at every sample. Surrogate recoveries were within acceptance limits throughout (fluorobenzene 99–108 %; naphthalene-d8 68–104 %; nonane 61–92 %).

Outcome. All TPH C_{10}–C_{50}, naphthalene and BTEX results were below Criterion B. Taking the 12 TPH determinations with non-detects at the 100 mg kg^{-1} reporting limit, the post-test mean was 216 mg kg^{-1} (184 mg kg^{-1} excluding the field duplicate), corresponding to an abatement of 88–90 % relative to the PC3 pre-treatment value of 1,780 mg kg^{-1}. Xylenes fell from 48 to ≤ 0.7 mg kg^{-1} and ethylbenzene from 13.2 to ≤ 0.5 mg kg^{-1}.

Residual methylnaphthalene exceedances (shaded). The demonstration test did not achieve compliance for the methylnaphthalene isomers at borehole FV1: 1-methylnaphthalene reached 1.6 mg kg^{-1} in the FV1-E7 field duplicate and 1.0 mg kg^{-1} at FV1-E8, and 2-methylnaphthalene reached 1.7 mg kg^{-1} at FV1-E8, against a Criterion B of 1 mg kg^{-1} for each isomer. These residuals were among the considerations supporting progression to a full-scale, multi-well campaign.

Field-duplicate agreement. The FV1-E7 field duplicate returned 567 mg kg^{-1} against 321 mg kg^{-1} for the primary sample, a relative percent difference of 55 %. Comparable divergence was observed for the naphthalene series (1-methylnaphthalene 1.6 vs 0.9 mg kg^{-1}) and, in the full-scale validation campaign, at FS11-E4 (381 vs 192 mg kg^{-1}, 66 %; Table S3). This is characteristic of short-range heterogeneity in a residually contaminated sandy matrix and quantifies the uncertainty attached to any point-scale pre/post comparison at this site (Section 4.5).

Table S5. Treated effluent monitoring

Concentrations measured in treated effluent at the compliance-verification tank, downstream of the diffused-air aeration unit and upstream of discharge to the City of Quebec combined sewer, over the 10 discrete monitoring events of the campaign (14 August – 28 November 2025). These are post-treatment values: they characterize discharge compliance, not the contaminant mass extracted from the subsurface, which was not quantified (Section 4.5). The maximum authorized discharge rate of 6.94 L min^{-1} was respected at all times.

Sampling date	Benzene	Toluene	Ethyl-benzene	Xylenes	Naphtha-lene	TPH C_{10}–C_{50}	Campaign phase
	(µg L^{-1})	(µg L^{-1})	(µg L^{-1})	(µg L^{-1})	(µg L^{-1})	(µg L^{-1})	
14-08-2025	<0.3	<1.0	<0.3	<1.0	1.9	<100	Start-up, water rinse
15-08-2025	<0.3	11.7	<0.3	<1.0	<0.1	<100	Start-up, water rinse
22-08-2025	0.6	6.1	4.9	22.8	0.6	<100	First surfactant injections
28-08-2025	0.5	5.4	3.8	19.7	0.4	<100	First surfactant injections
08-09-2025	40.3	179	53.2	248	5.3	<100	Peak mobilization (1st)
26-09-2025	0.8	4.0	0.5	3.0	0.2	<100	Rinse phase
03-10-2025	1.2	5.2	0.7	3.2	0.5	<100	Rinse phase
17-10-2025	14.5	17.7	2.3	26.4	3.3	<100	Renewed injection
14-11-2025	18.0	111	34.0	186	1.7	981	Peak mobilization (2nd)
28-11-2025	3.0	5.0	3.0	<1.0	<0.1	203	Final rinse / shutdown
Municipal discharge limit	50 [i]	200	160	370	200 [j]	15,000	—

[i]Site-specific limit set in the municipal discharge permit. [j] Naphthalene is regulated within the sum of polycyclic aromatic hydrocarbons (ΣPAH = 200 µg L^{-1}); the value shown is the naphthalene contribution alone.

Values reported as "<" are below the reported detection limit for the corresponding determination. TPH C_{10}–C_{50} remained below the 15,000 µg L^{-1} permit limit at every event: it was below the 100 µg L^{-1} reporting limit up to and including 17 October and was detected only during the second mobilization peak, at 981 µg L^{-1} on 14 November and 203 µg L^{-1} on 28 November. The per-event series is also plotted in Figure 5B of the main text.

Pattern. Effluent BTEX did not decline monotonically over the campaign. Two mobilization peaks were recorded — 8 September 2025 (toluene 179, xylenes 248, ethylbenzene 53.2, benzene 40.3 µg L^{-1}) and 14 November 2025 (toluene 111, xylenes 186, ethylbenzene 34.0, benzene 18.0 µg L^{-1}) — separated by a period of low concentrations during rinse

operations, with a smaller intermediate response on 17 October. Each peak followed the resumption of washing-solution injection in a part of the well network not previously treated, consistent with the stop-and-go protocol described in Section 2.5. All values remained below their respective permit limits, the closest approach being toluene at 90 % of its limit on 8 September 2025. The two peaks also differed in composition: TPH C_{10}–C_{50} was below the reporting limit throughout the first peak and was recovered only during the second, consistent with the lower aqueous solubility and stronger sorption of the heavier fractions.

Table S6. Fluid delivery log and pore-volume normalization

Injected fluid volumes for the full-scale campaign (12 August – 5 December 2025), normalized to the pore volume of the treated interval. The pore volume is computed for the 235 m^2 area of interest over the 1.5 m screened thickness of the treatment wells (3.0–4.5 m bgs), using an assumed effective porosity of 0.30 representative of the fine-to-medium sand characterized in Section 3.1 (93.7 % sand, 6.0 % silt + clay, 0.3 % gravel). Porosity was not measured directly at this site; the value adopted is a literature-typical figure for this texture and the derived pore volumes should be read as order-of-magnitude normalizations.

Item	Washing solution	Rinse water	Total	Note
Dedicated injection wells P23, P18, PC2 (m^3)	10.8	10.8	21.6	3.6 m^3 each
Stop-and-go cycles, all wells (m^3)	4.5	20.4	24.9	Diluted solution alternating with rinse
Total injected (m^3)	15.3	31.2	46.5	12 Aug – 5 Dec 2025 (115 d)
Pore volumes at porosity 0.30 (PV)	0.14	0.30	0.44	235 m^2 × 1.5 m × 0.30 = 105.8 m^3
Pore volumes at porosity 0.25–0.40 (PV)	0.11–0.17	0.22–0.35	0.33–0.53	Sensitivity; 88.1–141.0 m^3
Extracted, treated and discharged (m^3)	—	—	≈ 810	Operating records; not metered

Injected volumes were recorded at the injection manifold. No cumulative flow totalizer was installed on the extraction line; the extracted volume (≈ 810 m^3, about 17 times the injected volume) was compiled from the site operating records and is consistent with the start-up extraction rate (≈ 10 m^3 per 24 h) and with the discharge-permit upper bound (≈ 1,150 m^3 over 115 days) (Section 2.7 of the main text). No contaminant mass balance could be closed (Section 4.5). The porosity-sensitivity rows show that the comparison of Section 4.1 does not depend on the assumed value of 0.30.

Context. The delivered dose of 0.14 pore volumes of washing solution is at the low end of the published field-scale range (approximately 2.4 PV at Hill Air Force Base and approximately 1.5 PV at Bachman Road). The comparison is discussed in Section 4.1 of the main text.

Table S7. Soil and groundwater properties

Properties of the treated interval (3.0–4.5 m bgs) and of groundwater, compiled from the site investigations. Pre-treatment groundwater results are from accredited laboratories; field parameters were measured with a multi-parameter probe (Section 2.4 of the main text).

Parameter	Location / date	Value
**Soil — treated interval**		
Texture	Composite sample	Sand 93.7 %; silt + clay 6.0 %; gravel 0.3 %
Saturated hydraulic conductivity	PB1, PB2, PO2, PF2, PF3; 2 Oct 2024	2.66–6.35 × 10^{-5} m s^{-1} (n = 5)
Moisture content	Post-treatment cores; 5 Dec 2025	14.4–17.7 % (n = 7)
Effective porosity	—	0.30 assumed (sensitivity 0.25–0.40; Table S6)
Not measured	—	Bulk density, pH, organic carbon, CEC, specific surface area, water repellency, water retention
**Groundwater — before treatment**		

Parameter	Location / date	Value
PO-24-02 (sample / field duplicate)	27 Jun 2024	TPH C_{10}–C_{50} 1,000 / 1,000; benzene 130 / 140; toluene 50 / 52; ethylbenzene 460 / 480; xylenes 1,200 / 1,300; naphthalene 130 / 110 μg L^{-1}
PC3, low-flow (before demonstration test)	23 Sep 2024	TPH C_{10}–C_{50} 213 / 224 μg L^{-1}; SO_4^{2-} 149 mg L^{-1}; NH_4^+ 0.64 and TKN 1.0 mg N L^{-1}; NO_3^- + NO_2^- < 0.20 mg N L^{-1}; total P 0.40 mg L^{-1}; BOD_5 < 2 and COD 19 mg L^{-1}
PO2, outside injected zone	6–7 Mar 2025	pH 7.44–7.64; salinity 0.75–0.93 PSU
Other	All monitoring wells	LNAPL not detected; DO, ORP and temperature not recorded under undisturbed conditions
**Washing-solution tracers**		
Wells A and B (0.5–1.0 m from PC2)	6–7 Mar 2025	pH 10.4–12.6; salinity up to 12.6 PSU
Pumped treatment wells	17–23 Nov 2025	EC 0.24–6.6 mS cm^{-1}; pH 6.2–10.6
Municipal rinse water (reference)	18 Nov 2025	EC 0.23 mS cm^{-1}; pH 7.5; salinity 0.11 PSU

Table S8. Quebec generic soil criteria compared with CCME soil quality guidelines (mg kg^{-1})

Quebec generic criteria A, B and C (B = residential land use) are those applied in this study [10]. CCME values are for residential/parkland land use and coarse-grained soil [2,11]. Soil remediation is regulated provincially in Canada; CCME values are national benchmarks that provinces may adopt or adapt.

Parameter	Quebec A	Quebec B	Quebec C	CCME, residential/parkland, coarse-grained soil
TPH C_{10}–C_{50} (Quebec) / PHC fractions (CCME)	100	700	3,500	F1 (C_6–C_{10}) 30; F2 (>C_{10}–C_{16}) 150; F3 (>C_{16}–C_{34}) 300; F4 (>C_{34}) 2,800 [a]
Benzene	0.2	0.5	5	0.0095 (10^{-6}) / 0.030 (10^{-5}) [b]
Toluene	0.2	3	30	0.37 [b]
Ethylbenzene	0.2	5	50	0.082 [b]
Xylenes (total)	0.4	5	50	11 [b]
Naphthalene	0.1	5	50	0.6 [c]
1-Methylnaphthalene, 2-methylnaphthalene, 1,3-dimethylnaphthalene (each)	0.1	1	10	No CCME guideline

[a] Canada-Wide Standard for Petroleum Hydrocarbons in Soil, Tier 1, surface soil [2]; F1 is determined after subtraction of BTEX. The CCME fractions are not directly comparable with the Quebec C_{10}–C_{50} parameter, which spans F2 to F4. Subsoil and fine-grained soil levels differ for some fractions [2].

[b] Canadian Soil Quality Guidelines (2004), surface soil [11]. Benzene: 0.0095 mg kg^{-1} at an incremental cancer risk of 10^{-6} and 0.030 mg kg^{-1} at 10^{-5} (coarse subsoil: 0.011 and 0.030 mg kg^{-1}); the risk level is set by each jurisdiction. Fine-grained soil: benzene 0.0068, toluene 0.08, ethylbenzene 0.018 and xylenes 2.4 mg kg^{-1}. Toluene, ethylbenzene and xylene values are identical for surface soil and subsoil.

[c] 1997 provisional guideline, retained in the 2010 CCME update for PAHs [11]; a value of 0.013 mg kg^{-1} for the protection of freshwater life may be applied site-specifically.
